# Sports-based mental health promotion for adolescents in rural Nepal: A pilot cluster-randomised controlled trial

**DOI:** 10.1371/journal.pgph.0005991

**Published:** 2026-05-18

**Authors:** Kelly Rose-Clarke, Damodar Rimal, Indira Pradhan, Nabin Lamichhane, Gerard Abou Jaoude, John Hodsoll, Joanna Morrison, Nagendra Prasad Luitel

**Affiliations:** 1 Institute for Global Health, University College London, London, United Kingdom; 2 Transcultural Psychosocial Organization Nepal, Kathmandu, Nepal; 3 Institute of Psychiatry, Psychology and Neuroscience, Kings College London, London, United Kingdom; Institute of Public Health Bengaluru, INDIA

## Abstract

Adolescent mental health disorders are a leading contributor to the global burden of disease, particularly in low- and middle-income countries where access to care is limited. Sport offers a promising platform for mental health promotion, but evidence from rigorous evaluations in these settings is lacking. In Nepal we tested the feasibility of a mental health promotion intervention comprising football, dance and martial arts coaching and community engagement through *melas* (community celebrations), home visits and consultation with community advisory groups. We conducted a parallel-group, two-arm superiority pilot cluster-randomised controlled trial with 1:1 allocation ratio. The study area was four community clusters (~1000 population per cluster). We conducted pre-intervention (n = 440) and post-intervention (n = 403) cross-sectional surveys of all adolescents aged 12–19 living in the clusters. The intervention was implemented for 10 months and open to all aged 12–19. Control was sport as usual. We collected data according to pre-specified progression criteria and compared trial arms post-intervention for wellbeing, depression, anxiety, self-esteem, self-efficacy, emotion regulation, social support and functional impairment. Among adolescents in intervention clusters 110/224 (49.11%) attended ≥5 sessions. Younger age and belonging to the least privileged caste group was associated with attendance. In 146/191 (76.44%) sessions, coaches demonstrated ≥75% of activities at satisfactory/superior level according to a fidelity checklist. We found only a small and uncertain difference in wellbeing between arms (effect size 0.05, 95% confidence interval -0.45 to 0.64) but moderate evidence towards improved depression (-0.32, -0.67 to 0.02), anxiety (-0.27, -0.61 to 0.06) and social support from a significant other (0.41, 0.05 to 0.77) in the intervention arm. The average implementation cost per session was $67.63 ($0.07 per adolescent in the community per session). Findings suggest the intervention is feasible and might help to reduce mental health inequities. Progression to a phase III trial is warranted.

## Introduction

Mental health disorders, especially depression and anxiety, are leading causes of the global burden of disease among adolescents. An estimated one in seven adolescents aged 10–19 experiences mental disorders with negative consequences for their social and emotional development, relationships and educational attainment, and long-term effects on physical health and longevity [1–3]. Because most adolescents live in low- and middle-income countries (LMICs) the burden of mental disorders is greatest in these settings. Existing mental health care for adolescents in LMICs is inadequate and a priority for research and funding [4,5].

Mental health promotion and prevention are promising intervention approaches in LMICs, in addition to treatment [6]. Mental health promotion aims to strengthen positive aspects of mental health and psychosocial wellbeing; prevention aims to reduce the risk of developing a mental disorder. Universal prevention is a type of primary prevention which targets the whole population on the basis that everyone is likely to receive some benefit, in contrast to selective and indicated primary prevention targeting sub-populations and individuals at risk of developing or showing early signs of a disorder [7]. Among children and adolescents aged 0–18 years in LMICs, promotion/universal prevention may reduce depressive symptoms (mean difference MD -3.04, 95% confidence interval CI -6 to -0.08) and anxiety symptoms (MD -2.27, CI -3.13 to -1.41) and improve quality of life (standardised MD -0.25, CI -0.39 to -0.11) compared to usual care [6].

Physical activity has been used as a strategy for mental health promotion/universal prevention with adolescents and was highlighted in the World Health Organization (WHO) and United Nations Children Fund (UNICEF) Helping Adolescents Thrive Toolkit [8]. Systematic reviews and meta-analyses report potential protective effects of physical activity for adolescent mental health, including increased self-esteem, self-efficacy, cognitive performance and resilience, and reduced symptoms of depression and attention deficit hyperactivity disorder [9–11]. Proposed pathways through which physical activity improves mental health include neurobiological (e.g., activation of the hypothalamic-pituitary-adrenal axis), psychosocial (improved self-concept and competence) and behavioural mechanisms (improved sleep duration, timing and quality) triggered by increased physical activity [12]. For young people, physical activity almost invariably involves sport which may offer benefits above and beyond physical activity alone. These are related to the social aspects of playing sport and include belonging and companionship, self-esteem, self-control, mastery and social support [13].

Sports interventions for adolescent mental health are promising. They are potentially low-cost, popular with adolescents and scalable through clubs and schools. Different sports are likely to have different benefits for mental health because of their various physical and social requirements, and particular sports could theoretically be selected to suit an individual’s mental health needs [13]. However, there are also important drawbacks. There are few randomised controlled trials of sports-based mental health promotion/prevention interventions for adolescents in LMICs, and many are at high risk of bias [14–17]. Sport can be exclusive, for example cultural norms and menstruation are barriers to girls’ participation in sport in some settings [18]. Contextual factors such as the physical and social environment in which an individual engages in sport, how and by whom it is facilitated potentially influence the effect of sport on mental health [19]. Programmes focusing on performance and competition can increase stress with negative effects on mental health, especially in settings with a history of conflict [20]. This highlights the need for sports-based interventions to be tailored to the communities within which they are implemented.

We sought to address the lack of high-quality evidence for sports-based adolescent mental health promotion in LMICs and develop an intervention that responds to adolescents’ needs and priorities in a rural setting in Nepal. The SMART (Sports-based Mental heAlth pRomotion for adolescenTs) intervention was informed by qualitative research with adolescents, caregivers, teachers and sports coaches to identify community needs, preferences and priorities, mapping sport for development programmes in Nepal and a review of randomised trials of sports-based interventions for adolescent mental health to consolidate local and global learning [21]. SMART involved sports coaching and community engagement activities delivered over 10 months. Through a pilot cluster-randomised controlled trial, we sought to assess the feasibility of SMART and inform decisions to progress to a phase III trial.

## Methods

### Ethics statement

The study was approved by Nepal Health Research Council (3088) and King’s College London Research Ethics Committee (RESCM-22/23–27152, RESCM-22/23–33427).

We obtained written permission from the local municipality to implement the research.

Adolescent participants aged 18 or 19 provided written consent for themselves. Adolescent participants aged 12–17 were asked for assent and their caregivers provided written consent. Additional information regarding the ethical, cultural and scientific considerations specific to inclusivity in global research is included in the Supporting Information (S1 File).

### Study design and setting

The pilot trial took place between March 2023 and February 2024. It was prospectively registered on the International Standard Randomized Controlled Trial Number (ISRCTN) registry (ISRCTN15973986) and ClinicalTrials.gov (NCT05394311) and the protocol was published [21]. The design was a parallel group, two-arm pilot cluster-randomised controlled trial with 1:1 allocation ratio and cross-sectional surveys pre and post intervention. A trial steering committee reviewed and approved the trial protocol and statistical analysis plan.

The trial was conducted in Bardiya district on the Indian border in the plains region of western Nepal in Province 5. The district population size is 459,900 (female-to-male ratio of 1.12:1) of whom 9.4% are aged 10–14 and 9.9% are 15–19 [22]. More than half the population is from the Tharu ethnic group and speak Tharu as their first language [23]. Agriculture is the main source of income but labour migration out of the district is common: 28% of households have at least one member abroad [24]. A third (32.8%) of the married population aged 10 and above were married before they were 18. Among women, 44.1% were married before the age of 18 [22]. Three quarters (76.9%) of the population aged five and above can read and write [22].

The trial was conducted in four clusters in Bardiya. Each cluster was a community with a population of around 1000. We mitigated the risk of contamination by purposively selecting clusters separated by distance or natural boundaries such as jungle or farmland. We selected clusters that were ethnically diverse to improve the generalisability of our findings to other parts of Nepal.

### Study participants

We conducted census-like surveys before the intervention (baseline, 25^th^ January 2023–9^th^ May 2023) and after (endline, 5^th^ January 2024–16^th^ March 2024). Survey participants were adolescents aged 12–19 residing in the study clusters. We excluded adolescents who were not permanent residents (e.g., they were visiting family). We anticipated movement of adolescents into and out of the clusters due to migration, marriage and education. Our statistical analysis plan therefore accounted for any differences between baseline and endline samples.

### Randomisation and masking

We randomised clusters prior to the baseline survey. One pair of clusters was rural and relatively inaccessible; the other pair was semi-urban and close to health services. We therefore randomised the clusters in pairs to ensure one rural and one semi-urban cluster were allocated to each arm. Randomisation was conducted using SPSS software by a researcher who was not part of the trial team and unfamiliar with the study setting. We could not conceal allocation from study participants and intervention staff due to the nature of the intervention. Research assistants who conducted surveys worked from a separate office, were not informed about the intervention, were unaware of intervention staff and remained masked to allocation.

### Intervention

We implemented SMART in one intervention cluster from the 28^th^ of March 2023 until the 25^th^ of January 2024, and in the second from the 10^th^ of May 2023 until the 23^rd^ of February 2024. SMART comprised football, martial arts (karate and taekwondo) and dance coaching sessions led by local coaches, and community engagement activities. Each session was 90 minutes and comprised welcome, review and objective setting, warm up and breathing, skill development, cool-down, reflection and mood check activities. In this way, we integrated sports and mental health promotion activities into each session to reduce mental health stigma and optimise adolescent engagement. Sports coaches were trained by a Nepali psychosocial counsellor according to the SMART intervention manual. Training drew on the WHO’s competency-based *Foundational Helping Skills* – a module to build basic psychosocial skills - and covered topics including empathy, verbal and non-verbal communication, listening, safeguarding, management of high-risk cases and self-care. Coaches were also trained on group facilitation skills and basic first aid. Coaches received weekly online supervision from a psychosocial counsellor in Kathmandu. Approximately every ten sessions the supervisor travelled to Bardiya to observe sessions in person, provide in person supervision and conduct refresher training.

Community engagement activities comprised: (i) in each intervention cluster, three *melas* (community celebrations) incorporating sports demonstrations by coaches and adolescents, competitions, prize-giving and community drama to celebrate adolescents’ achievements and raise awareness about issues affecting adolescent mental health; (ii) visits by coaches to adolescents’ homes to talk to adolescents and their families about the intervention and provide support to facilitate adolescents’ participation in the sessions; (iii) coaches meeting community groups to explain and mobilise support for the intervention and; (iv) two meetings in each cluster with an adolescent and a community advisory group to identify and resolve emerging issues and inform ongoing intervention implementation.

### Comparator

Adolescents in the control arm were free to participate in sports activities and access health services as usual. We trained health workers in the municipality on the WHO mental health Gap Action Programme Intervention Guide, contextualised for Nepal. We provided information in control and intervention clusters about how to access these trained health workers. At the end of the trial we provided sports equipment to the municipality.

### Data collection

Four research assistants (two males and two females aged 24–31) from the local area conducted baseline and endline surveys. Prior to the baseline survey, research assistants received two weeks’ training on interviewing techniques, the survey tools, ethics and data quality. We assessed inter-rater reliability among the research assistants to ensure consistency and uniformity in data collection. Clusters were surveyed in the same order in baseline and endline surveys. In each cluster, research assistants went to every household to enquire as to whether any eligible adolescents lived there. Where adolescents were identified, research assistants described the study to the caregiver and adolescent and provided the information sheet and consent form. Research assistants conducted the interview at a time and place that was convenient for the adolescent, usually in their home. Interviews were conducted in Nepali. Survey questions included questions on demographics, education, household income, daily functioning, participation in sport, mental health and wellbeing. Adolescents with elevated depression or anxiety symptoms were given information about local services, a free mental health hotline number and self-care techniques. Adolescents disclosing suicidal risk behaviour were referred to a psychosocial counsellor. Data were collected using tablets programmed with Kobo Toolbox. To help ensure high quality data, we used automated skip patterns and consistency checks. The research team kept records of any errors and these were corrected during data analysis.

### Process evaluation data

We conducted a mixed methods process evaluation informed by our theory of change and Medical Research Council guidance for process evaluation [25]. We sought to assess intervention implementation, mechanisms of change and contextual factors affecting implementation, mechanisms and outcomes [25,26]. Quantitative process evaluation methods and analyses are described here. We mainly used qualitative data to explore mechanisms and contextual factors, and these analyses will be reported in a future publication.

Under implementation, we assessed fidelity to explore whether SMART was delivered as per the manual, and reach defined as the extent to which the target population came into contact with the intervention [25]. To assess fidelity, the intervention coordinator assessed coaches during coaching sessions using a 20-item fidelity checklist of session activities and foundational helping skills (Table 1). Response options were coded 0 “not attempted”, 1 “needs improvement”, 2 “satisfactory” and 3 “superior”. A total of 191/316 sessions (60.44%) were observed across the two intervention clusters (58 football sessions, 67 dance sessions and 66 martial arts sessions).

**Table 1:** Activities and skills included in the Fidelity Checklist.

Activity/skill	Definition
1. Welcomes	Welcomes adolescents and outlines aims and objectives for the session
2. Recaps	Recaps at start of the session on what adolescents learned in the last session, discusses how it connects to psychosocial skills and adolescents' lives outside coaching (home/school) and how they applied learning for positive change in their lives, and asks what changes did adolescents see
3. Rules	Discusses/reminds about ground rules and how to play safely
4. Warm-up	Conducts warm up
5. Relaxation	Conducts a relaxation or breathing exercise
6. Relating	Relates sports activities to psychosocial skills (cool down)
7. Mood check	Conducts mood check and discusses emotions
8. Reflects	Reflects on what adolescents have learned in the current session, connects it to psychosocial skills and their lives outside coaching (home/school), how they can apply learning for positive change in their lives
9. Builds confidence	Builds adolescents’ confidence by appreciating encouraging and praising adolescents of all abilities
10. Discusses emotions	Encourages the adolescents to discuss and/or acknowledge emotions, such as anger, sadness, guilt and shame at home or with friends. Normalises these emotions (e.g., says: It’s okay to feel angry about this situation; many people do)
11. Discusses behaviour	Observes and discusses how the adolescents can be affected by each other, taking examples from coaching sessions to illustrate how others' behaviour can affect us)
12. Problem solving	At the end of the session, asks the group: What did you learn today? What is your goal for the next session and how will you achieve it?
13. Helps	Points out how one person has helped another.
14. Listens	Listens first before offering any advice
15. Thanks	Encourages and thanks adolescents for contributing to the group discussion
16. Describes benefits of sport	Educates on how sport, sleep, nutrition and/or fitness can help to manage stress/mental health (cool-down)
17. Builds rapport	Works at establishing rapport and group cohesion
18. Shares time	Shares time fairly amongst group members
19. Focus	Keeps group focused
20. Summarises	Summarises the session, talks about the plan for the next session and closes the session effectively/happily

To assess reach, we collected data from coaching session attendance records including information about the gender, caste/ethnic group and socioeconomic status of adolescents who attended. We also identified any adolescents who attended the sessions living outside the intervention clusters. To assess contamination, we also identified any adolescents living in control clusters.

Process data were collated in a monthly report and reviewed by the trial team. During intervention implementation these reports informed strategies to promote attendance at coaching sessions, including home visits by coaches and tailoring sessions to engage older adolescents. Fidelity data were used by the supervisor to inform the content of refresher training for coaches during the intervention period.

### Outcomes

#### Feasibility outcomes and progression criteria.

We set a priori progression criteria related to: fidelity to the SMART manual (at least 75% of activities in each session completed at a satisfactory or superior level according to the fidelity checklist); exposure to the intervention (at least 30% of adolescents aged 12–19 living in the intervention clusters participate in at least five coaching sessions); adverse events (fewer than 10% of adolescents experience an untoward physical or psychological occurrence potentially related to the intervention); missing data (fewer than 15% missing items on mental health and wellbeing outcome measures at endline); and contamination across trial arms (fewer than 10% of adolescents living in control clusters participate in the intervention).

#### Trial outcomes.

The primary outcome was mental wellbeing measured using the 14-item Warwick Edinburgh Mental Wellbeing Scale (WEMWBS-14). Secondary outcomes were self-esteem measured with the Rosenberg Self-Esteem Scale, self-efficacy with the Generalised Self-Efficacy Scale, emotion regulation strategies with the Adolescent Emotion Regulation Strategies Questionnaire (AERSQ) and social support with the Multidimensional Scale of Perceived Social Support. We also measured depression and anxiety symptoms using the Patient Health Questionnaire 9-items modified for adolescents (PHQ-A) and the General Anxiety Disorder 7-item (GAD-7) respectively [27,28], and functional impairment with a locally developed tool [29]. The PHQ-A and GAD-7 have been translated, adapted and validated in Nepal [30].

Prior to the baseline survey we translated the WEMWBS-14, Rosenberg Self-Esteem scale, Schwarzer Self Efficacy tool and the AERSQ according to guidelines recommended for cross-cultural research [31]. This involved translation of the tool from English into Nepali by bilingual experts; review of the translation by Nepali mental health practitioners and researchers; 21 focus group discussions with adolescents aged 12–19 to explore their understanding of the scale items and to ensure cultural acceptability, relevance, and age-appropriate language; 33 cognitive interviews with adolescents to assess their interpretation of each item, identify any confusing or ambiguous wording, and refine the scale to enhance its clarity, comprehensibility, and cultural relevance; and back-translation by an external person who was not involved in the study. We used data from the baseline survey (n = 440) to assess the tools’ internal consistency: Cronbach’s alpha ranged from 0.78 (Rosenberg Self Esteem Scale) to 0.86 (Generalised Self Efficacy Scale). As our primary outcome, we further evaluated psychometric properties of the WEMWBS-14. Criterion validity (the relationship between WEMWBS-14 and potential predictors of adolescent wellbeing) was acceptable. WEMWBS-14 was negatively correlated with the PHQ-A (-0.19 95% confidence interval CI -0.28, -0.09), GAD-7 (-0.16 95% CI -0.16, -0.06) and functional impairment (-0.29 95% CI -0.38, -0.20), and positively correlated with self-efficacy (0.57 95% CI 0.50, 0.64), self-esteem (0.45 95% CI 0.37, 0.53) and social support (0.49 95% CI 0.41, 0.57). Test-retest reliability after two weeks in a sample of 50 adolescents aged 12–16 was also adequate (individual 0.72, 95% CI 0.56-0.83; average 0.84, 0.72-0.91).

In addition to the WEMWBS-14 there is a short form version which was developed to address poor fit to a Rasch model of some of the 14 items on Wave 12 of the Scottish Health Education Survey [32]. The motivation for fitting a Rasch model is that it allows the identification of a uniform interval scale where the sum score reflects the underlying latent trait, in this case mental wellbeing. Stewart-Brown et al reported that a seven-item version (WEMWBS-7) showed a better fit as an interval scale measure of mental wellbeing to the 14-item original [32]. We therefore included WEMWBS-7 as a secondary outcome in the trial.

### Sample size

The pilot trial was not powered to detect outcome differences between intervention and control arms. We included four clusters based on available resources which enabled us to pilot randomisation and intervention implementation across different sites. Based on national data, we estimated 17.4% of the population in the four clusters would be aged 12–19 which corresponds to 696 adolescents [21]. Assuming 20% of adolescents were living away at the time of the study, we estimated a sample size of 556.

### Analysis

We conducted data analyses according to a detailed, pre-specified statistical analysis plan approved by the Trial Steering Committee.

### Process data analysis

To assess fidelity to the SMART manual, we calculated the mean percentage of activities in each session completed at a satisfactory or superior level according to the fidelity checklist. In line with our progression criteria, we also calculated the number of sessions in which at least 75% of activities were completed at a satisfactory or superior level.

We examined attendance at coaching sessions including attendance by adolescents living within and outside intervention clusters, calculating the percentage of sessions attended by adolescents overall and by gender and caste/ethnic group. With consent, we linked attendance data with data collected from the same adolescent in baseline and endline surveys to assess the percentage and characteristics of adolescents from intervention clusters who participated in the coaching sessions. We defined intervention compliance as participating in five or more coaching sessions based on our a priori hypothesis that five sessions would be sufficient for adolescents to improve their psychosocial skills. We compared compliant versus non-compliant adolescents and used logistic regression to evaluate baseline predictors of compliance. Adverse events and severe adverse events were descriptively summarised by trial arm.

### Analysis of trial outcomes

For the SMART trial, the estimand was the cluster-level average treatment effect or group difference in wellbeing (measured using the WEMWBS scale) at 10 months post-randomisation for 12 – 19-year-old adolescents residing in the clusters randomised to the SMART intervention versus control. This target treatment effect is regardless of participation within the intervention and other potential intermediate events. We descriptively summarised baseline demographic and clinical variables for each of the two arms of the trial (and across trial arms) at the cluster and individual participant level. For the primary and secondary outcomes, we used a linear mixed model approach to adjust for the statistical dependency from clustering and repeated measures. We conducted a matched cohort analysis using a constrained baseline analysis to allow for inclusion of participants who completed either the baseline or follow-up survey only [33]. We did not adjust for covariates because randomisation is designed to balance both observed and unobserved confounders across groups, so an unadjusted analysis provides an unbiased estimate of the intervention effect. Also, this was a pilot trial with few clusters, and including multiple covariates could have led to overfitting and unstable estimates. We did not apply multiplicity adjustments for multiple outcomes because the analyses were exploratory and intended to inform the design of a future definitive trial. Predictors of missingness at endline were assessed in univariable analyses and multivariable analyses. Predictors were included in the primary analysis model if significant in multivariable analysis (here age at p < 0.05 level) to allow the inclusion of all participants with at least one measure under the assumption of missing at random. The primary analysis model also included fixed effects of treatment group (intervention or control), time (baseline or endline) and age and random effects of cluster, participant and time at the cluster level with the constraint of no group difference between the groups at baseline. Estimation was via restricted maximum likelihood. We calculated estimates of mean group differences with 95% confidence intervals and standardised effects sizes by dividing these estimates by the respective baseline standard deviation. To account for the small number of clusters which can lead to an increased risk of a type 1 error, we used the subcluster wild bootstrap.

The main analyses used an intent to treat approach, but we also assessed effects of intervention compliance through a per protocol analysis. We conducted analyses to examine whether effect size was moderated by gender and age. We identified potential interactions using the ratio of difference in means to standard error (SE), considering a difference in means two or more times greater than the SE as potentially important. This approach is often used as an exploratory first step to identify candidate moderators and is appropriate for this pilot trial which was not designed for definitive interaction testing.

We also examined intervention effect sizes according to different levels of exposure to SMART, creating a binary exposure variable to identify those participants who attended more than five sessions (based on our a priori hypothesis that participation in five sessions would be sufficient for adolescents to build their psychosocial skills [21]). The effect of level of intervention exposure (categorised as <5, 5–15, 16–35, > 35 sessions) was estimated by including the interaction between the exposure variable and intervention group. We used Stata 18 and R 4.3 for the main descriptive and inferential analyses.

### Cost analysis

We carried out a within-trial cost analysis of the SMART pilot from the provider perspective. As a pilot cost analysis, the primary aim was to understand indicative cost parameters to inform future scale-up and definitive evaluation, not to produce generalisable cost estimates. Resources and costs related to research were excluded from the analysis. We developed a costing tool in Microsoft Excel to estimate the annual cost of the intervention, the average cost per session and average cost per adolescent in community clusters. Project accounts, records and interviews with staff informed resource use and cost data analysed in the costing tool. Economic costs were estimated, which capture any donated goods or volunteer time by assigning current market value to such resources – for example, equivalent rental costs for sports facilities used free of charge during the project. Costs were categorised into start-up or implementation and by type of input (e.g., capital, staff, material), as well as allocated across intervention activities such as training and recruitment, melas or sports sessions. Where resources or costs were related to multiple activities, allocations were informed by interviews with project staff and staff time allocations. We consulted staff about their time allocations using interviewer-administered timesheets for three timepoints: manual and intervention development, setting up of the intervention and at the end of implementation. All costs were captured in Nepali Rupees and inflated to 2024, the base year of analysis, before converting to US dollars. Results in this paper are therefore presented in 2024 US dollars. A univariate sensitivity analysis investigated the impact on results of increasing or decreasing staff costs by 25%, which were the primary cost driver.

## Results

### Study population and baseline descriptives

**Fig 1:**
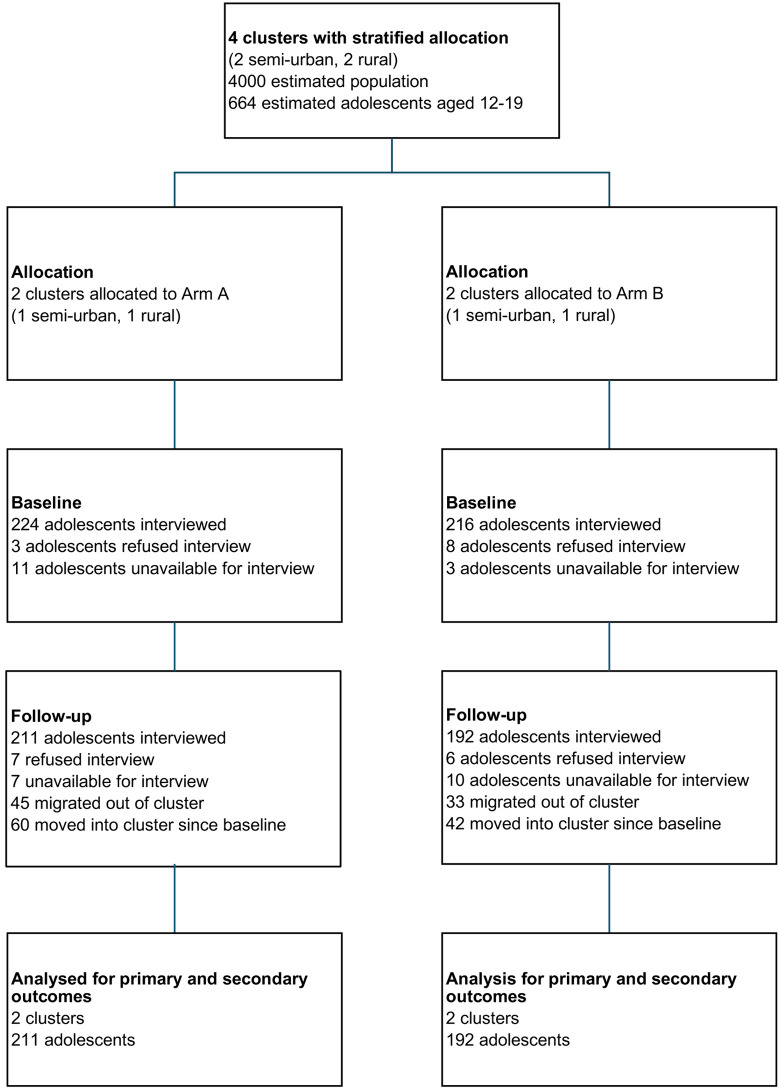
SMART participant CONSORT flow diagram.

Fig 1 is the trial consort diagram. 440 adolescents aged 12–19 completed the baseline survey: 224 (50.9%) in the intervention arm and 216 (49.1%) in the control arm. Trials arms were relatively balanced with regards to mean age, gender and religion (Table 2). Compared to intervention, in the control arm there was a higher proportion of adolescents belonging to the least privileged Dalit caste group (33.3% vs 23.7%) and fewer adolescents from wealthier households with sufficient income for 10–12 months (30.1% vs 42.4%).

**Table 2:** Socio-demographic characteristics of participants at baseline by trial arm.

Variables	Intervention (n = 224)	Control (n = 216)	Total (n = 440)
**Mean age (SD)**	14.9 (2.14)	14.7 (2.19)	14.8 (2.17)
**Gender (%)**			
Male	98 (43.75)	96 (44.44)	194 (44.09)
Female	126 (56.25)	120 (55.56)	246 (55.91)
**Caste/ethnic group (%)**			
Most privileged: Brahman, Chhetri, Thakur, Puri	93 (41.52)	74 (34.26)	167 (37.95)
More privileged: Janajati, Tharu, Yadav	78 (34.82)	70 (32.41)	148 (33.64)
Least privileged: Dalit	53 (23.66)	72 (33.33)	125 (28.41)
**Religion (%)**			
Hindu	199 (88.84)	186 (86.11)	385 (87.50)
Buddhist	9 (4.02)	7 (3.24)	16 (3.64)
Christian	11 (4.91)	21 (9.72)	32 (7.27)
Atheist	2 (0.89)	0	2 (0.45)
Other	3 (1.34)	2 (0.93)	5 (1.14)
**Education Level (%)**			
Primary	38 (16.96)	31 (14.35)	69 (15.68)
Lower Secondary	96 (42.86)	112 (51.85)	208 (47.27)
Secondary	90 (40.18)	73 (33.80)	163 (37.05)
**Currently studying (%)**			
No	17 (7.59)	11 (5.09)	28 (6.36)
Yes	207 (92.41)	205 (94.91)	412 (93.64)
**Income sufficiency (%)**			
0-3 months	12 (5.36)	17 (7.87)	29 (6.59)
4-6 months	87 (38.84)	92 (42.59)	179 (40.68)
7-9 months	15 (6.70)	22 (10.19)	37 (8.41)
10-12 months	95 (42.41)	65 (30.09)	160 (36.36)
Don’t know	15 (6.70)	20 (9.26)	35 (7.95)

Out of the 403 adolescents who participated in the endline survey, 102 (25.3%) were new survey participants who had not participated in the baseline survey. These new participants were younger, with a lower level of education and more likely to be from the Dalit caste group compared to the sample at baseline (S1 Table). Across trial arms, the proportion of new participants was relatively balanced (intervention 60/211, 28.4%; control 42/192, 21.9%). In the control arm there was a higher proportion of new participants from Janajati, Tharu and Yadav groups and fewer from the Dalit group compared to the intervention arm.

### Process evaluation

Findings from the process evaluation are presented against the progression criteria in Table 3 and discussed in the following sections.

**Table 3:** Summary of progression criteria and pilot trial findings.

Feasibility outcome	Progression criteria	Pilot trial findings
Fidelity to the intervention manual	At least 75% of activities in each session completed at a satisfactory/superior level according to a fidelity checklist.	In 146 of 191 (76.44%) coaching sessions observed, at least 75% of the activities were completed at a satisfactory or superior level.
Exposure to the intervention	At least 30% of adolescents aged 12–19 living in the intervention clusters participate in at least five coaching sessions.	Out of 224 participants living in the intervention arm at baseline, 110 (49.11%) participated in at least five coaching sessions
Adverse events	Fewer than 10% of adolescents experience an adverse event (i.e., an untoward physical or psychological occurrence which is potentially related to the intervention) or a severe adverse event (i.e., an adverse event that is life threatening, results in death and/or requires hospitalisation).	7/548 adolescents (1.28%) who participated in the intervention experienced an adverse event (fainting n = 3, minor injury n = 3, bee sting n = 1).
Missing data	Fewer than 15% missing items on mental health and wellbeing outcome measures at endline	0% of items on mental health and wellbeing outcome measures were missing at endline.
Contamination across trial arms	Fewer than 10% of adolescents living in the control clusters participated in the intervention.	1 adolescent (0.46%) living in the control arm participated in the coaching sessions.

#### Attendance.

548 adolescents attended at least one coaching session including 224 adolescents living in the study clusters and 324 adolescents living outside the clusters. Over two thirds (75/108, 69.4%) of adolescents aged 12–14 who participated in the baseline survey attended at least five coaching sessions compared to 30.2% (35/116) of 15–19-year-olds (Table 4). Only one adolescent (0.5%) living in the control arm participated in the coaching sessions. Across genders and age groups, a higher proportion of adolescents attended five or more football sessions (85/224, 38.0%) compared to dance (54/224, 24.1%) or martial arts (56/224. 25.0%).

**Table 4:** Baseline participants who attended at least five coaching sessions by gender and age.

Name of Sports	Gender		Age group		Total n=224 (%)
	Boys n=98 (%)	Girls n=126 (%)	12-14 Years n=108 (%)	15-19 Years n=116 (%)	
Any sport	50 (51.02)	60 (47.62)	75 (69.44)	35 (30.17)	110 (49.11)
Football	40 (40.82)	45 (35.71)	59 (54.63)	26 (22.41)	85 (37.95)
Martial Arts	26 (26.53)	30 (23.81)	46 (42.59)	10 (8.62)	56 (25.00)
Dance	16 (16.33)	38 (30.16)	47 (43.52)	7 (6.03)	54 (24.11)

Among baseline participants we explored predictors of attending coaching sessions (S2–S4 Tables). In multivariable analyses we found that younger age was associated with attendance, predicted marginal means (based on the model fitted in S4 Table) showed each year of age decreased the probability of attending five sessions by 8.7% (95%CI 11.3% to 6.7%) and belonging to the least privileged caste group increased likelihood of attendance by 21.3% (95% CI 34.3% to 8.4%) compared to other groups.

#### Intervention fidelity.

Across all sports, in 146 of 191 (76.4%) coaching sessions observed at least 75% of the activities were demonstrated at a satisfactory or superior level. Fidelity was highest in martial arts (54/66, 81.8% of sessions with at least 75% of activities at satisfactory/superior level) and lowest in dance (47/67, 70.2%). Fidelity improved over time: in the last two months of the intervention (Jan and Feb 2024) at least 75% of activities were performed at satisfactory/superior level in 28/28 (100%) sessions.

#### Adverse events.

Of the 548 adolescents participating in the intervention, seven experienced problems that were potentially related to the intervention and could be defined as adverse events (fainting n = 3, minor injury n = 3, bee sting n = 1).

#### Missing data.

Among 403 adolescents who participated in the endline survey, 0% of survey items were missing. We also explored missing data among adolescents who moved out of the study clusters during intervention implementation. Of the 440 baseline survey participants, 301 (68.4%) also participated in the endline survey. Endline data were not available for 139/440 (31.6%). In multivariable analysis, older age predicted missingness at endline (OR 1.64, 95% CI 1.44-1.87) but trial arm did not (S5–S7 Tables).

### Outcome analysis

**Fig 2:**
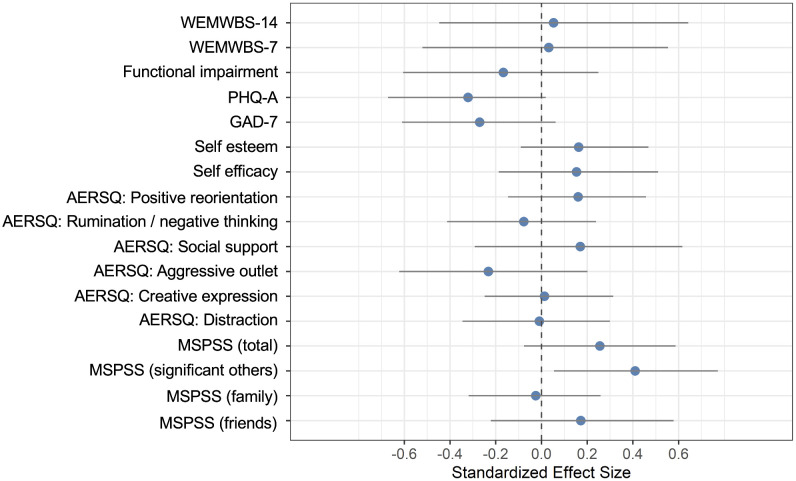
Outcomes and effect sizes.

Table 5 presents primary and secondary outcomes by trial arm at baseline and endline. Table 6 and Fig 2 present the mean difference in scores for primary and secondary outcomes at endline. For the primary outcome, mental wellbeing, there was no difference between mean scores at endline between intervention and control arms, assessed using 14- and 7-item versions of the WEMWBS (WEMWBS-14 effect size ES 0.05, 95% CI -0.45, 0.64; WEMWBS-7 ES 0.03; 95% CI -0.52, 0.55). Depression (ES -0.32 95% CI -0.67, 0.02) and anxiety symptoms (ES -0.27, 95% CI -0.61 - 0.06) were lower in the intervention arm compared to control. Social support was higher in the intervention arm, especially social support from a significant other (ES 0.41, 95% CI 0.05, 0.77).

**Table 5:** Descriptive statistics of outcomes at individual level at baseline and endline.

		Intervention		Control		ICC^a^ (95% CI)
		N	Mean (SD)	N	Mean (SD)	
**Mental well-being (WEMWBS-14)**	Baseline	224	50.2 (7.8)	216	51.0 (8.2)	
	Endline	211	51.5 (8.2)	192	51.4 (8.8)	0.01 (0.00, 0.21)
**Mental well-being (WEMWBS-7)**	Baseline	224	24.2 (4.2)	216	24.3 (4.6)	
	Endline	211	24.8 (4.4)	192	24.8 (4.9)	.00 (0.00, 1.00)
**Self-esteem (RSE)**	Baseline	224	31.7 (3.6)	216	31.9 (4.0)	
	Endline	211	32.3 (3.9)	192	31.8 (4.2)	0.00 (0.00, 0.00)
**Self-efficacy (SSES)**	Baseline	224	28.9 (5.1)	216	29.7 (4.9)	
	Endline	211	29.7 (4.7)	192	29.4 (5.2)	0.00 (0.00, 0.00)
**Emotion regulation: AERSQ**						
*AERSQ: Positive reorientation*	Baseline	224	10.1 (3.2)	216	10.5 (3.0)	
	Endline	211	10.7 (3.1)	192	10.4 (3.1)	0.00 (0.00, 0.00)
*AERSQ: Rumination/ negative thinking*	Baseline	224	6.3 (3.3)	216	7.1 (3.5)	
	Endline	211	5.7 (3.6)	192	6.5 (3.5)	.01 (0.00, 0.14)
*AERSQ: Social Support*	Baseline	224	7.9 (3.1)	216	8.4 (3.6)	
	Endline	211	8.5 (3.6)	192	8.3 (3.3)	0.01 (0.00, 0.16)
*AERSQ: Aggressive outlet*	Baseline	224	2.1 (2.7)	216	2.6 (3.2)	
	Endline	211	1.5 (2.2)	192	2.3 (3.1)	.02 (0.00,.14)
*AERSQ: Creative expression*	Baseline	224	2.4 (2.9)	216	2.5 (2.7)	
	Endline	211	2.9 (3.3)	192	2.9 (3.5)	0.00 (0.00, 0.00)
*AERSQ: Distraction*	Baseline	224	6.4 (2.3)	216	6.8 (2.4)	
	Endline	211	6.4 (2.4)	192	6.6 (2.6)	0.00 (0.00, 0.00)
**Depression (PHQ-A)**	Baseline	224	5.8 (4.0)	216	5.8 (4.6)	
	Endline	211	5.0 (4.2)	192	5.9 (4.1)	.01 (.00,.15)
**Anxiety (GAD-7)**	Baseline	224	5.3 (3.9)	216	5.1 (3.9)	
	Endline	211	4.2 (3.7)	192	4.9 (4.1)	.01 (.00,.19)
**Functional impairment**	Baseline	224	3.7 (3.4)	216	3.3 (3.3)	
	Endline	211	3.1 (2.8)	192	3.4 (3.0)	.01 (.00,.16)
**MSPSS: Total**	Baseline	224	44 (7.9)	216	46 (8.1)	
	Endline	211	45.3 (8.2)	192	45.0 (8.4)	.00 (.00,.00)

^a^ICC intracluster correlation coefficient

**Table 6:** Mean difference between trial arms at endline and standardised effect size.

Outcome	Mean difference^1^	95% CI^2^	Effect size^3^	95% CI^4^
WEMWBS-14	0.34	-2.79, 3.92	0.05	-0.45, 0.64
WEMWBS-7	0.10	-1.58, 1.69	0.03	-0.52, 0.55
Functional impairment	-0.42	-1.50, 0.62	-0.17	-0.61, 0.25
Depression (PHQ-A)	-1.08	-2.21, 0.07	-0.32	-0.67, 0.02
Anxiety (GAD-7)	-0.84	-1.79, 0.19	-0.27	-0.61, 0.06
Self-esteem	0.5	-0.28, 1.41	0.16	-0.09, 0.47
Self-efficacy	0.6	-0.78, 2.01	0.15	-0.19, 0.51
AERSQ: Positive reorientation	0.4	-0.35, 1.14	0.16	-0.15, 0.46
AERSQ: Rumination/negative thinking	-0.21	-1.15, 0.65	-0.08	-0.41, 0.24
AERSQ: Social Support	0.46	-0.76, 1.70	0.17	-0.29, 0.62
AERSQ: Aggressive outlet	-0.52	-1.40, 0.45	-0.23	-0.62, 0.20
AES: Creativity expression	0.03	-0.60, 0.78	0.01	-0.25, 0.31
AES: Distraction	-0.02	-0.65, 0.56	-0.01	-0.35, 0.30
MSPSS: Total	1.64	-0.52, 3.73	0.26	-0.08, 0.59
MSPSS: Significant others	1.14	0.15, 2.14	0.41	0.05, 0.77
MSPSS: Family	-0.06	-0.72, 0.60	-0.03	-0.32, 0.26
MSPSS: Friends	0.48	-0.64, 1.57	0.17	-0.22, 0.58

^1^Mean difference: Estimated mean difference between groups; ^2 CI^: Confidence interval; ^3^Effect size: Mean difference standardized by baseline standard deviation; ^4 CI^: Bootstrapped confidence interval.

**Fig 3:**
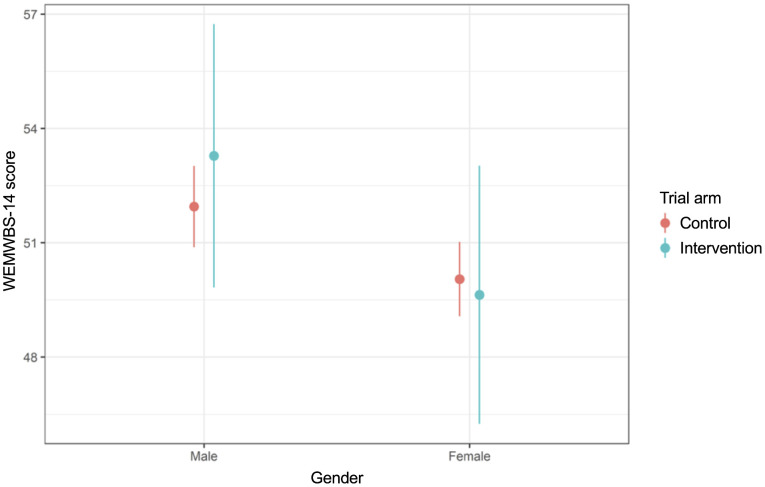
Interaction plot for gender and mental wellbeing (WEMWBS-14).

**Fig 4:**
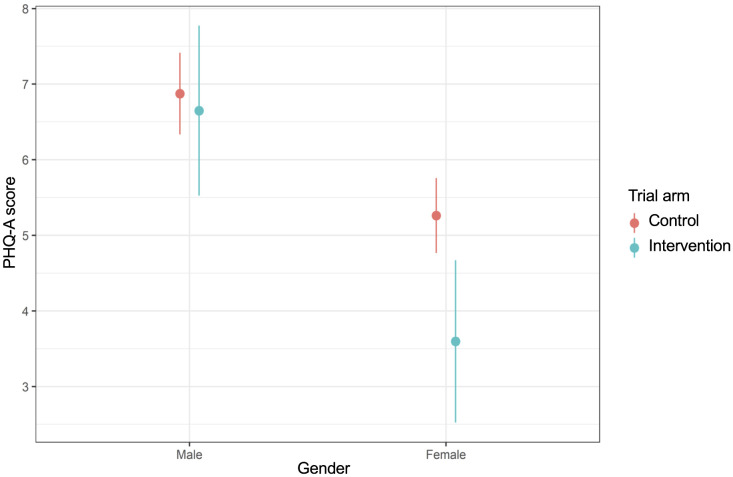
Interaction plot for gender and depressive symptoms (PHQ-A).

**Fig 5:**
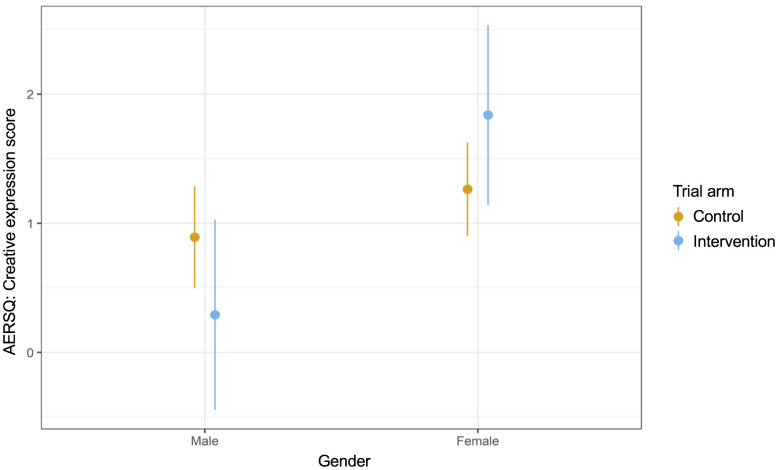
Interaction plot for gender and creativity (AERSQ).

We conducted analyses to explore whether the effect size was moderated by gender (S8 Table) and age (S9 Table). We found some evidence of an increase in mental wellbeing for boys in the intervention arm compared to control, but not for girls (Fig 3). Depressive symptoms decreased for girls in the intervention arm versus control but not for boys (Fig 4). Concerning emotion regulation strategies, in the intervention arm relative to control there was a decrease in creativity for boys but an increase for girls (Fig 5). We found no evidence of moderation by age (S9 Table).

We explored potential indirect effects of the intervention with the subgroup of endline survey participants who attended fewer than five coaching sessions. We found evidence suggesting depressive and anxiety symptoms reduced irrespective of adolescents’ participation in coaching sessions (Table 7, depression ES -0.40 95% CI -0.85, 0.03, anxiety ES -0.33 95% CI -0.76, 0.09) although the reduction was not significant.

**Table 7:** Trial outcomes among endline survey participants in the intervention arm who participated in fewer than five coaching sessions versus control.

Outcome	Mean difference^1^	Effect size^2^
WEMWBS-14	-0.04 (-3.71, 3.69)	-0.01 (-0.57, 0.56)
WEMWBS-7	-0.05 (-2.14, 1.82)	-0.02 (-0.68, 0.59)
Functional impairment	-0.71 (-1.92, 0.48)	-0.29 (-0.78, 0.18)
Depression (PHQ-A)	-1.32 (-2.84, 0.11)	-0.40 (-0.85, 0.03)
Anxiety (GAD-7)	-1.02 (-2.31, 0.25)	-0.33 (-0.76, 0.09)
Self-esteem	0.56 (-0.51, 1.72)	0.18 (-0.16, 0.56)
Self-efficacy	0.32 (-1.30, 1.95)	0.08 (-0.33, 0.48)
AERSQ: Positive reorientation	-0.40 (-1.31, 0.56)	-0.16 (-0.55, 0.22)
AERSQ: Rumination/negative thinking	-0.14 (-1.24, 0.92)	-0.05 (-0.47, 0.33)
AERSQ: Social Support	0.16 (-1.14, 1.54)	0.06 (-0.44, 0.57)
AERSQ: Aggressive outlet	-0.59 (-1.65, 0.48)	-0.24 (-0.73, 0.22)
AES: Creativity expression	-0.41 (-1.34, 0.48)	-0.17 (-0.56, 0.20)
AES: Distraction	-0.16 (-0.91, 0.51)	-0.10 (-0.48, 0.27)
MSPSS: Total	0.95 (-1.40, 3.42)	0.15 (-0.21, 0.55)
MSPSS: Significant others	0.78 (-0.36, 1.93)	0.28 (-0.13, 0.70)
MSPSS: Family	-0.12 (-1.04, 0.74)	-0.05 (-0.47, 0.32)
MSPSS: Friends	0.30 (-1.07, 1.59)	0.10 (-0.39, 0.59)

1 Mean difference (95% CI): Estimated mean difference between groups.

2 Effect size: Mean difference standardized by baseline standard deviation.

We explored the effect of increasing the intervention “dose” by fitting a model predicting the number of sessions based on baseline variables in the intervention arm and using this model to predict the number of sessions participants in the control arm would have done if they had been in the intervention arm. We estimated the intervention effect for different levels of attendance (<5, 5–15, 16–35, > 35 sessions). We found potential effects of increasing dose for depression, anxiety, functional impairment, self-esteem and social support from a significant other (Table 8).

**Table 8:** Effect of dose on trial outcomes.

No. sessions	Mean difference	95% CI	p value
***Mental wellbeing (WEMWBS-14)***			
<5	3.41	-0.80, 7.63	0.11
5-15	-0.36	-4.25, 3.54	0.855
16-35	-0.31	-4.20, 3.58	0.874
>35	-1.65	-4.66, 1.35	0.279
***Functional impairment***			
<5	-0.67	-2.07, 0.72	0.337
5-15	0.23	-0.89, 1.35	0.681
16-35	1.49	0.11, 2.88	0.035
>35	0.02	-1.13, 1.17	0.975
***Depression (PHQ-A)***			
<5	-0.87	-2.92, 1.18	0.398
5-15	0.49	-1.11, 2.09	0.541
16-35	2.12	0.42, 3.81	0.015
>35	1.8	0.31, 3.28	0.018
***Anxiety (GAD-7)***			
<5	-0.7	-2.50, 1.10	0.439
5-15	0.53	-1.20, 2.27	0.542
16-35	1.51	-0.22, 3.23	0.085
>35	1.48	0.07, 2.89	0.04
***Self-esteem***			
<5	0.03	-1.95, 2.01	0.973
5-15	-0.39	-2.02, 1.23	0.631
16-35	1.13	-0.61, 2.87	0.198
>35	-1.35	-2.68, -0.03	0.045
***Self-efficacy***			
<5	1.1	-1.30, 3.50	0.362
5-15	-0.11	-2.00, 1.78	0.906
16-35	-0.45	-2.79, 1.89	0.701
>35	-0.35	-2.04, 1.33	0.679
***MSPSS: Total***			
<5	-1.25	-5.27, 2.76	0.534
5-15	-2.05	-4.86, 0.76	0.151
16-35	-1.55	-5.29, 2.20	0.41
>35	-1.04	-3.87, 1.79	0.467
***MSPSS: Significant others***			
<5	0.43	-1.41, 2.26	0.642
5-15	-1.34	-2.73, 0.05	0.058
16-35	-0.76	-2.57, 1.05	0.405
>35	-1.51	-2.76, -0.26	0.018
***MSPSS: Family***			
<5	0.48	-0.92, 1.88	0.496
5-15	0.14	-0.99, 1.26	0.812
16-35	-0.31	-1.75, 1.13	0.664
>35	-0.25	-1.30, 0.81	0.644
***MSPSS: Friends***			
<5	-1.34	-2.93, 0.24	0.094
5-15	-0.88	-2.11, 0.35	0.159
16-35	-0.25	-1.66, 1.17	0.726
>35	0.62	-0.60, 1.85	0.316

### Cost analysis results

The combined economic cost of intervention set-up and implementation, over the entire project duration, was US$ 71,497 (S10 Table). Over half of this was accounted for by coaching session facilitation (40%) and mela activities (24%). The average annual cost of implementation and set-up combined was US$ 30,642, equivalent to US$ 33.10 per adolescent in the community, US$ 96.97 per session held and US$ 0.10 per adolescent in the community per session. Implementation costs over the project duration were US$ 49,867, or 70% of the total cost including set-up. The average annual implementation cost was US$ 21,371, equivalent to US$ 23.08 per adolescent in the community, US$ 67.63 per session held and US$ 0.07 per adolescent in the community per session. This translates to US$ 0.35 per adolescent in the community for a minimum of five sessions. Staff were the main cost driver, accounting for an average of 58.8% of overall costs, 64.8% of session facilitation costs and 41.9% of mela costs. Increasing staff costs by 25% in the sensitivity analysis resulted in average implementation costs of US$ 26.70 per adolescent in the community, US$ 78.21 per session held and US$ 0.08 per adolescent in the community per session – while a 25% reduction resulted in costs of US$ 19.47 per adolescent in the community, US$ 57.05 per session held, and US $0.06 per adolescent in the community per session.

## Discussion

We found that SMART, a sports-based mental health intervention for adolescents in rural Nepal, was feasible and low-cost. We demonstrated that the intervention was acceptable to adolescents, evidenced by almost half of all adolescents in the intervention arm participating in at least five coaching sessions during the 10-month implementation period. We met progression criteria concerning exposure to the intervention, adverse events, missing data and contamination across trial arms. We report initial trends for depression and anxiety outcomes suggestive of benefits for mental health that could be significant in a fully powered phase III trial.

In terms of intervention fidelity to the SMART manual, we observed that in 76.4% of sessions observed at least 75% of activities were completed at a satisfactory or superior level. This is lower than our progression criteria which specified that 75% of activities “in each session” (i.e., 100% of sessions) should be completed at satisfactory/superior level. With hindsight, this was overly ambitious and did not account for coaches’ improvement over time. Prior to the study, coaches had been trained to focus on improving sports performance and found it initially challenging to change their mindset and reorient coaching to integrate mental health promotion. During the intervention, we used fidelity data from the sessions to identify areas for improvement and focused coaches’ refresher training on these areas. Coaches’ performance improved with time, demonstrating the importance of these data for quality improvement as well as fidelity assessment.

We found that SMART was more popular among younger than older adolescents. Currently studying did not predict attendance in multivariable analyses independently of age, suggesting that participation was not associated with school attendance. Qualitative process data suggest that it was more socially challenging for older adolescents to learn a new skill in a relatively public place. Older adolescents who were already familiar with the sport often felt that the intervention skill level was too basic for them (manuscript in preparation). We excluded adolescents younger than 12 because they mainly attend basic schools (lower basic grades 1–5, upper basic grades 6–8) and our formative research suggested adolescents attending secondary school (grades 9–12) did not want to play with them, but during the intervention many of these younger adolescents petitioned coaches to join. Future implementation of SMART could be targeted at adolescents aged 12–14 and explore the acceptability of involving 10- and 11-year-olds.

Gender did not predict participation in the intervention despite formative research findings suggesting multiple challenges including menstruation, gendered household norms, harassment, and concepts that sport participation was of little value for girls and could affect their marriageability. We purposively sought to address barriers to girls’ participation by recruiting a male and female coach for each sport, offering dance sessions because girls said they were interested in learning dance and it may be more acceptable to families compared to football and martial arts; in sessions, valuing team work and personal growth rather than strength and ability; and visiting homes to build relationships with adolescents’ families and address their concerns. For coaching sessions, adolescents could wear any clothes that made them feel comfortable, as girls may feel uncomfortable wearing shorts and t-shirts. Girls and boys trained together but for football we separated teams by gender during games. There were no penalties for late arrival or infrequent attendance, enabling girls and boys with household responsibilities to participate when they could, and allowing girls to take time out while menstruating if they wished. We also addressed and discussed the ways that gender affects participation in sport in our community dramas.

We conducted a pilot trial that was not powered to detect intervention effects. Compared to control, we did not observe improvements in mental wellbeing among adolescents in the intervention arm. However, our findings show trends suggestive of benefits for depression and anxiety which should be evaluated in a fully powered trial. Because SMART is implemented at a population level, even a small effect could potentially equate to a large and clinically meaningful impact at scale. Possible explanations for improvements in depression and anxiety but not wellbeing could relate to intervention mechanisms. We hypothesised that SMART would promote wellbeing through pathways including improved self-efficacy, self-esteem, social support and stress management [21]. Whilst we saw improvements in social support (significant other) there was minimal change in self-efficacy and self-esteem, and we did not assess stress management. Plausibly intervention mechanisms related to prevention of mental ill health rather than promotion of wellbeing, through targeted support for at-risk groups. For example, participation in coaching sessions was highest among Dalits, a caste group at increased risk of mental health problems who may have benefitted via mechanisms that prevent depression and anxiety or alleviate sub-threshold symptoms [34]. Another possibility is that among adolescents in the intervention arm, there was a higher uptake of mental health services because the intervention raised awareness. We did not ask adolescents about service use in the endline survey, but this would be important data to collect in a future trial.

Another plausible explanation for the null wellbeing results could be due to the survey tool. There is increasing recognition that concepts of wellbeing are influenced by culture [35]. For example, a study found concepts of happiness in settings with more traditional values (emphasising religion, deference to authority and family values) relate more to religion and psychological definitions of happiness compared to settings with secular-rational values (less emphasis on religion, family and authority) where definitions more often relate to relationships, work, living standards and health [36,37]. In Nepal, having all basic needs met, freedom of movement and being able to perform traditional rituals have been identified as important components of wellbeing [38,39] and educational attainment is a central focus of Nepali adolescent aspirational models [40]. We used the WEMWBS to measure wellbeing in this study. The scale was developed in the UK and based on a tool from New Zealand [41]. It covers eudemonic and hedonic aspects of wellbeing according to ancient Greek theories of wellbeing. The extent to which these concepts are relevant to our study population compared to Hindu and Buddhist concepts and local values remains unclear. Although our psychometric evaluation of the WEMWBS established that the tool was accurate and meaningful, deeper engagement with adolescents on underlying concepts of wellbeing is needed.

Our findings suggest a potential indirect effect of SMART on depression and anxiety among adolescents who did not participate in five coaching sessions, although this needs to be tested in a fully powered trial. There are plausible pathways through which an indirect effect could arise. First, adolescents may not need to participate in five sessions to benefit from SMART; one, two, three or four sessions could be sufficient. Second, adolescents who did not participate in the intervention could still have attended melas and benefitted from the exposure to mental health awareness and advocacy activities at these events. Third, adolescents who participated in coaching sessions may have shared the skills they learned with their friends who did not participate. Fourth, some coaching sessions were held in public spaces which could have created a safer environment for adolescents to socialise even if they were not participating in the sessions. Fifth, there is emerging evidence that watching sport and/or supporting a team can improve mental health independent of any effects due to physical participation. For example, being part of a fan community can increase an individual’s sense of belonging [42,43]. Watching sport can provide a distraction from stress and worry, and watching others overcome adversity and succeed in their sport can be inspiring and motivating [44]. However, longitudinal studies on this topic examining cause and effect are needed, as is research on the effects of community sport spectatorship, spectatorship among adolescents and spectatorship in non-Western settings.

The average cost per adolescent in the community per SMART coaching session was US$ 0.07. There are few comparative cost analyses of mental health promotion interventions in LMICs. A systematic review of cost effectiveness of mental health prevention and promotion interventions found no cost evaluations of child and adolescent interventions from LMICs, though among those conducted in high-income countries the majority demonstrated good value for money [45]. Universal school-based socio-emotional learning to improve mental health is included in the WHO menu of cost-effective mental health interventions with an estimated cost of < I$ 100,000 per one million population in LMICs and an average cost-effectiveness ratio (I$/ healthy life year gained) of 1000–5000 [46]. In India, a multi-component whole-school health promotion intervention (SEHER) with benefits for mental health cost $15 per student when delivered by lay counsellors and $7.4 when delivered by teachers (though only the lay counsellor-delivered model improved mental health) [47]. Whilst it is not possible to predict costs of SMART at scale, coaches could potentially cover a larger population than the pilot trial if we adopt a training of trainers approach where coaches support local people to become lay coaches. This would substantially reduce costs and promote cost-effectiveness.

Findings from the study have implications for policies and plans in Nepal. For example, SMART aligns with and supports multiple strategic objectives of the *National Mental Health Strategy and Action Plan 2020* including provision of non-stigmatising community-based mental health support and increasing public awareness on mental health [48]. SMART provides a potential model for inclusive, community-based mental health promotion and awareness, addressing priority areas of the *National Adolescent Health and Development Strategy 2018* (Strategic Objective 7.3) and the *National Youth Policy 2015* (Strategy 3.14) [49]. Local engagement and partnership with policy makers ahead of a phase III trial will help to ensure SMART aligns with school and municipality-led mental health and sports initiatives. Coordination across health, education, youth and sports ministries will be important to identify potential support and funding for scale up and sustainability beyond the trial.

### Limitations

We worked in two intervention clusters in one rural district, limiting the generalisability of our findings to other parts of Nepal although we selected communities with diverse caste/ethnic groups. We randomised clusters before conducting the baseline survey so that we could identify a suitable location for the intervention office and begin recruitment and training of sports coaches. Although this potentially increased the risk of selection bias this was offset by conducting a census-like survey where every adolescent aged 12–19 living in the clusters was included. Moreover, participants and recruiters (research assistants) were masked to allocation, research assistants worked from a separate office to the intervention team and were unaware of the nature of the intervention. In developing the phase III trial protocol, we will revisit the feasibility of randomising after the baseline survey. Although contamination was limited to one adolescent from the control arm participating in the coaching, more adolescents (n = 324) living outside the intervention clusters (but not in control clusters) participated than those living in the intervention clusters (n = 224). We were unable to collect data outside the intervention clusters and therefore could not assess the impact of SMART on these adolescents. In a phase III trial, expanding the size of the cluster unit to capture the full effect of the intervention is plausible, whilst respecting administrative boundaries and mitigating the risk of contamination.

## Conclusion

SMART is a feasible and low-cost sports-based mental health intervention for adolescents in Nepal. Our pilot trial has generated sufficient evidence to proceed to a fully powered phase III trial to assess impact and cost-effectiveness. Intervention activities should target younger adolescents, and a process evaluation is needed to explore mechanisms for direct and indirect intervention effects and how these interact with different adolescent groups and contexts. Future studies should also examine Nepali adolescents’ understandings of mental wellbeing and the relevance of this concept in the context of a sports-based mental health promotion intervention.

## Supporting information

S1 File Inclusivity in global research. (DOCX)

S1 Table Socio-demographic characteristics of adolescents who participated in endline but not baseline. (DOCX)

S2 Table Univariable analyses of baseline socio-demographic predictors of attending five or more coaching sessions. (DOCX)

S3 Table Univariable analyses of baseline mental health predictors of attending five or more coaching sessions. (DOCX)

S4 Table Multivariable analysis of baseline predictors of attending five or more coaching sessions (n = 224). (DOCX)

S5 Table Univariable analysis of sociodemographic predictors of missing data at endline. (DOCX)

S6 Table Univariable analysis of mental health predictors of missing data at endline. (DOCX)

S7 Table Multivariable analysis of predictors of missing data at endline (n = 440). (DOCX)

S8 Table Moderation of effect size by gender. (DOCX)

S9 Table Moderation of effect size by age category (16–19 and 12–15 years). (DOCX)

S10 Table Cost of implementation and set up of SMART. (DOCX)

S1 Text KCL ethics application (development and baseline). (PDF)

S2 Text KCL ethics application (implementation and endline). (PDF)
