# Supplementary material for: Sports-based mental health promotion for adolescents in rural Nepal: A pilot cluster-randomised controlled trial
Source: PLOS Glob Public Health. 2026 May 18;6(5):e0005991. doi: 10.1371/journal.pgph.0005991 (PMC13183228; doi:10.1371/journal.pgph.0005991)
Supplement: S1 Table — (DOCX) [file pgph.0005991.s002.docx]

**S1 Table: Socio-demographic characteristics of adolescents who participated in endline but not baseline**

| **Characteristic** | **Intervention (n=60)** | **Control**  **(n=42)** | **Total (n=102)** |
| --- | --- | --- | --- |
| **Age** |  |  |  |
| Mean age (SD) | 13.6 (2.16) | 13.6 (2.39) | 13.6 (2.24) |
| Age group (%) |  |  |  |
| Younger adolescent (12 - 15 years) | 46 (76.7) | 32 (76.2) | 78 (76.5) |
| Older adolescent (16 - 19 years) | 14 (23.3) | 10 (23.8) | 24 (23.5) |
| **Gender** |  |  |  |
| Male | 27 (45.0) | 23 (54.8) | 50 (49.0) |
| Female | 33 (55.0) | 19 (45.2) | 52 (51.0) |
| **Caste/ethnic group** |  |  |  |
| More privileged caste groups: Brahman, Chhetri, Thakur, Puri | 22 (36.7) | 14 (33.3) | 36 (35.3) |
| Less privileged caste groups: Janajati, Tharu, Yadav | 13 (21.7) | 16 (38.1) | 29 (28.4) |
| Least privileged caste groups: Dalit | 25 (41.7) | 12 (28.6) | 37 (36.3) |
| **Religion** |  |  |  |
| Hindu | 54 (90.0) | 37 (88.1) | 91 (89.2) |
| Buddhist | 1 (1.7) | 1 (2.4) | 2 (2.0) |
| Christian | 4 (6.7) | 4 (9.5) | 8 (7.8) |
| Atheist | 1 (1.7) | 0 (0.0) | 1 (1.0) |
| Other | **-** | **-** | **-** |
| **Education Level** |  |  |  |
| Primary | 33 (55.0) | 22 (52.4) | 55 (53.9) |
| Lower secondary | 13 (21.7) | 12 (28.6) | 25 (24.5) |
| Secondary | 14 (23.3) | 8 (19.0) | 22 (21.6) |
| **Income sufficiency** |  |  |  |
| 0-3 months | 3 (5.0) | 1 (2.4) | 4 (3.9) |
| 4-6 months | 30 (50.0) | 19 (45.2) | 49 (48.0) |
| 7-9 months | 5 (8.3) | 2 (4.8) | 7 (6.9) |
| 10-12 months | 17 (28.3) | 15 (35.7) | 32 (31.4) |
| Don’t know | 5 (8.3) | 5 (11.9) | 1. 9.8) |

- Participants who participated in the endline survey but not the baseline were younger, with a lower level of education and more likely to be from the Dalit caste group compared to the sample at baseline. In the intervention arm there was a higher percentage of endline only participants from the Dalit caste group and lower percentage from Janajati, Tharu and Yadav groups compared to control.
