# Supplementary material for: Sports-based mental health promotion for adolescents in rural Nepal: A pilot cluster-randomised controlled trial
Source: PLOS Glob Public Health. 2026 May 18;6(5):e0005991. doi: 10.1371/journal.pgph.0005991 (PMC13183228; doi:10.1371/journal.pgph.0005991)
Supplement: S2 Table — (DOCX) [file pgph.0005991.s003.docx]

**S2 Table: Univariable analyses of baseline socio-demographic predictors of attending five or more coaching sessions**

| **Characteristic** | **N** | **Attended less than five coaching sessions**  n = 145^1^ | **Attended 5 or more coaching sessions**  n = 139^1^ | **p-value**^2^ |
| --- | --- | --- | --- | --- |
| **Age** | 284 | 16.00 (14.00, 17.00) | 13.00 (12.00, 15.00) | <0.001 |
| **Gender** | 284 |  |  | 0.2 |
| Male |  | 58 (40%) | 67 (48%) |  |
| Female |  | 87 (60%) | 72 (52%) |  |
| **Do you usually live with your mother?** | 284 |  |  | 0.002 |
| No |  | 46 (32%) | 22 (16%) |  |
| Yes |  | 99 (68%) | 117 (84%) |  |
| **Do you usually live with your father?** | 284 |  |  | 0.7 |
| No |  | 80 (55%) | 74 (53%) |  |
| Yes |  | 65 (45%) | 65 (47%) |  |
| **Are you currently studying?** | 284 |  |  | <0.001 |
| No |  | 22 (15%) | 2 (1.4%) |  |
| Yes |  | 123 (85%) | 137 (99%) |  |
| **Caste** | 284 |  |  | 0.063 |
| Least privileged caste groups:  Dalit |  | 31 (21%) | 47 (34%) |  |
| Less privileged caste groups: Janajati, Tharu, Yadav |  | 51 (35%) | 42 (30%) |  |
| More privileged caste groups: Brahman, Chhetri, Thakur, Puri |  | 63 (43%) | 50 (36%) |  |
| **Hindu** | 284 |  |  | 0.5 |
| No |  | 14 (9.7%) | 17 (12%) |  |
| Yes |  | 131 (90%) | 122 (88%) |  |
| **Income sufficient for** | 284 |  |  | 0.3 |
| 0-3 month |  | 8 (5.5%) | 7 (5.0%) |  |
| 4-6 month |  | 55 (38%) | 62 (45%) |  |
| 7-9 month |  | 7 (4.8%) | 13 (9.4%) |  |
| 10-12 month |  | 65 (45%) | 47 (34%) |  |
| Don't know |  | 10 (6.9%) | 10 (7.2%) |  |
| **Play sport** | 224 |  |  | 0.014 |
| No |  | 58 (51%) | 38 (35%) |  |
| Yes |  | 56 (49%) | 72 (65%) |  |
| Unknown |  | 31 | 29 |  |
| **Dance** | 224 |  |  | 0.2 |
| No |  | 85 (75%) | 74 (67%) |  |
| Yes |  | 29 (25%) | 36 (33%) |  |
| Unknown |  | 31 | 29 |  |
| ^1^ n (%); Median (IQR) | | | | |
| ^2^ Fisher’s exact test; Wilcoxon rank sum test; Pearson’s Chi-squared test | | | | |

- In univariable analyses, sociodemographic predictors of attending five or more coaching sessions at p < 0.2 were younger age, living with mother, studying, caste group and playing sport.
