# Supplementary material for: Sports-based mental health promotion for adolescents in rural Nepal: A pilot cluster-randomised controlled trial
Source: PLOS Glob Public Health. 2026 May 18;6(5):e0005991. doi: 10.1371/journal.pgph.0005991 (PMC13183228; doi:10.1371/journal.pgph.0005991)
Supplement: S3 Table — (DOCX) [file pgph.0005991.s004.docx]

**S3 Table: Univariable analyses of baseline mental health predictors of attending five or more coaching sessions**

| **Characteristic** | **N** | **Attended less than five coaching sessions,**  **N = 145^1^** | **Attended five or more coaching sessions,**  **N = 139^1^** | **p-value^2^** |
| --- | --- | --- | --- | --- |
| Mental wellbeing (WEMWBS-14) | 224 | 50 (45, 55) | 50 (45, 55) | 0.8 |
| Mental wellbeing (WEMWBS-7) | 224 | 21.5 (19.4, 24.1) | 21.5 (19.4, 24.1) | 0.7 |
| Depression (PHQ-A) | 224 | 5.0 (3.0, 8.0) | 5.5 (3.0, 8.0) | 0.6 |
| Anxiety (GAD-7) | 224 | 4.0 (2.0, 7.0) | 5.0 (3.0, 7.0) | 0.11 |
| Functional impairment | 224 | 3.0 (1.0, 5.0) | 3 (1.0, 5.0) | 0.3 |
| Self-efficacy | 224 | 28.0 (25.0, 33.0) | 28.5 (25.2, 33.0) | 0.3 |
| Self-esteem | 224 | 32.0 (29.2, 34.0) | 31.0 (30.0, 34.0) | 0.7 |
| AERSQ: Positive reorientation | 224 | 10.0 (8.2, 13.0) | 10.0 (8.0, 12.0) | 0.6 |
| AERSQ: Rumination/negative thinking | 224 | 6.0 (4.0, 8.0) | 6.0 (4.0, 9.0) | 0.4 |
| AERSQ: Social Support | 224 | 8.0 (6.0, 10.0) | 8.0 (6.0, 10.0) | 0.7 |
| AERSQ: Aggressive outlet | 224 | 1.00 (0.00, 2.00) | 2.00 (0.00, 4.00) | 0.062 |
| AES: Creativity expression | 224 | 1.00 (0.00, 3.00) | 2.00 (0.00, 5.00) | 0.052 |
| AES: Distraction | 224 | 7.00 (5.00, 7.00) | 6.00 (5.00, 8.00) | >0.9 |
| MSPSS: Total | 224 | 44 (39, 49) | 45 (38, 50) | 0.8 |
| MSPSS: Significant others | 224 | 14.5 (12.0, 16.0) | 14.0 (12.0, 16.0) | 0.2 |
| MSPSS: Family | 224 | 16.00 (14.25, 18.75) | 17.00 (15.00, 19.00) | 0.15 |
| MSPSS: Friends | 224 | 14.0 (11.0, 16.0) | 14.0 (11.0, 17.0) | 0.7 |

^1^ Median (IQR)

^2^ Wilcoxon rank sum test

- In univariable analyses, mental health predictors of attending five or more coaching sessions at p < 0.2 were anxiety, aggressive outlet, creativity expression and family support.
