# Supplementary material for: Sports-based mental health promotion for adolescents in rural Nepal: A pilot cluster-randomised controlled trial
Source: PLOS Glob Public Health. 2026 May 18;6(5):e0005991. doi: 10.1371/journal.pgph.0005991 (PMC13183228; doi:10.1371/journal.pgph.0005991)
Supplement: S4 Table — (DOCX) [file pgph.0005991.s005.docx]

**S4 Table: Multivariable analysis of baseline predictors of attending five or more coaching sessions (n=224)**

|  | **Dose** | | |
| --- | --- | --- | --- |
| **Predictors** | **Odds ratio** | **95% CI** | **p** |
| (Intercept) | 379.18 | 7.15,  22972.69 | **0.004** |
| Age | 0.62 | 0.51,  0.74 | **<0.001** |
| Cohabits with mother | 1.55 | 0.68,  3.58 | 0.294 |
| Currently studying | 2.43 | 0.52,  17.93 | 0.304 |
| Less privileged caste groups: Janajati, Tharu, Yadav | 0.39 | 0.16,  0.90 | **0.030** |
| More privileged caste groups: Brahman, Chhetri, Thakur, Puri | 0.27 | 0.11,  0.59 | **0.002** |
| Play sport | 1.07 | 0.55,  2.08 | 0.836 |
| Anxiety (GAD-7) | 1.07 | 0.98,  1.18 | 0.116 |
| AERSQ: Aggressive outlet | 0.96 | 0.85,  1.09 | 0.553 |
| AERSQ: Creative expression | 1.01 | 0.90,  1.13 | 0.849 |
| MSPSS: Family | 1.01 | 0.90,  1.14 | 0.822 |
| Cluster | 1.43 | 0.76,  2.68 | 0.264 |

- We included variables in a multivariable analysis of predictors of attendance if significant in univariable analyses at p<0.2 level (S2 and S3 Tables). In multivariable analysis, age and caste group predicted attendance at p<0.05 level.
