# Supplementary material for: Sports-based mental health promotion for adolescents in rural Nepal: A pilot cluster-randomised controlled trial
Source: PLOS Glob Public Health. 2026 May 18;6(5):e0005991. doi: 10.1371/journal.pgph.0005991 (PMC13183228; doi:10.1371/journal.pgph.0005991)
Supplement: S5 Table — (DOCX) [file pgph.0005991.s006.docx]

**S5 Table: Univariable analysis of sociodemographic predictors of missing data at endline**

| **Characteristic** | **N** | **Non missing**, N = 301^1^ | **Missing**, N = 139^1^ | **p-value**^2^ |
| --- | --- | --- | --- | --- |
| **Arm** | 440 |  |  | 0.6 |
| Intervention |  | 151 (50%) | 73 (53%) |  |
| Control |  | 150 (50%) | 66 (47%) |  |
| **Age** | 440 | 14.00 (12.00, 16.00) | 16.00 (15.00, 18.00) | <0.001 |
| **Gender** | 440 |  |  | 0.6 |
| Male |  | 135 (45%) | 59 (42%) |  |
| Female |  | 166 (55%) | 80 (58%) |  |
| **Do you usually live with your mother?** | 440 |  |  | 0.008 |
| No |  | 57 (19%) | 42 (30%) |  |
| Yes |  | 244 (81%) | 97 (70%) |  |
| **Do you usually live with your father?** | 440 |  |  | 0.4 |
| No |  | 168 (56%) | 72 (52%) |  |
| Yes |  | 133 (44%) | 67 (48%) |  |
| **Are you currently studying?** | 440 |  |  | <0.001 |
| No |  | 8 (2.7%) | 20 (14%) |  |
| Yes |  | 293 (97%) | 119 (86%) |  |
| **Caste** | 440 |  |  | 0.5 |
| Least privileged caste groups:  Dalit |  | 91 (30%) | 34 (24%) |  |
| Less privileged caste groups: Janajati, Tharu, Yadav |  | 98 (33%) | 50 (36%) |  |
| More privileged caste groups: Brahman, Chhetri, Thakur, Puri |  | 112 (37%) | 55 (40%) |  |
| **Hindu** | 440 |  |  | 0.8 |
| No |  | 37 (12%) | 18 (13%) |  |
| Yes |  | 264 (88%) | 121 (87%) |  |
| **Income sufficient for** | 440 |  |  | 0.8 |
| 0-3 month |  | 20 (6.6%) | 9 (6.5%) |  |
| 4-6 month |  | 117 (39%) | 62 (45%) |  |
| 7-9 month |  | 27 (9.0%) | 10 (7.2%) |  |
| 10-12 month |  | 111 (37%) | 49 (35%) |  |
| Don't know |  | 26 (8.6%) | 9 (6.5%) |  |
| **Play sport** | 440 |  |  | 0.007 |
| No |  | 119 (40%) | 74 (53%) |  |
| Yes |  | 182 (60%) | 65 (47%) |  |
| **Dance** | 440 |  |  | 0.12 |
| No |  | 203 (67%) | 104 (75%) |  |
| Yes |  | 98 (33%) | 35 (25%) |  |

- The table presents findings from univariable analysis of sociodemographic predictors of missingness. Findings here suggest associations between missingness and older age, not living with mother, not studying, not playing sport and not dancing at the p<0.2 level.
