# Supplementary material for: Sports-based mental health promotion for adolescents in rural Nepal: A pilot cluster-randomised controlled trial
Source: PLOS Glob Public Health. 2026 May 18;6(5):e0005991. doi: 10.1371/journal.pgph.0005991 (PMC13183228; doi:10.1371/journal.pgph.0005991)
Supplement: S6 Table — (DOCX) [file pgph.0005991.s007.docx]

**S6 Table: Univariable analysis of mental health predictors of missing data at endline**

| **Mental health outcome** | **N** | **Non missing**, N = 301^1^ | **Missing**, N = 139^1^ | **p-value**^2^ |
| --- | --- | --- | --- | --- |
| WEMWBS-14 | 440 | 51 (46, 55) | 50 (44, 57) | 0.7 |
| WEMWBS-7 | 440 | 21.5 (20.0, 24.1) | 21.5 (18.9, 25.0) | 0.7 |
| Depression (PHQ-A) | 440 | 5.0 (3.0, 8.0) | 6.0 (2.0, 9.0) | 0.4 |
| Anxiety (GAD-7) | 440 | 4.0 (2.0, 7.0) | 5.0 (2.0, 8.0) | 0.2 |
| Functional Impairment | 440 | 3.0 (1.0, 5.0) | 3.0 (1.0, 4.0) | 0.5 |
| Self-efficacy | 440 | 28.0 (25.0, 33.0) | 30.0 (27.0, 33.0) | 0.038 |
| Self-esteem | 440 | 32.0 (30.0, 34.0) | 31.0 (29.0, 34.5) | >0.9 |
| AERSQ: Positive reorientation | 440 | 10.0 (8.0, 12.0) | 11.0 (9.0, 13.0) | 0.088 |
| AERSQ: Rumination/negative thinking | 440 | 6.0 (4.0, 9.0) | 7.0 (5.0, 10.0) | 0.073 |
| AERSQ: Social support | 440 | 8.0 (6.0, 10.0) | 9.0 (6.0, 11.0) | 0.2 |
| AERSQ: Aggressive outlet | 440 | 1.00 (0.00, 4.00) | 1.00 (0.00, 3.50) | 0.4 |
| AERSQ: Creativity expression | 440 | 2.00 (0.00, 4.00) | 1.00 (0.00, 4.00) | 0.3 |
| AERSQ: Distraction | 440 | 7.00 (5.00, 8.00) | 7.00 (5.00, 8.00) | 0.3 |
| MSPSS: Total | 440 | 45 (40, 51) | 46 (39, 51) | 0.7 |
| MSPSS: Significant others | 440 | 15.0 (12.0, 17.0) | 15.0 (12.0, 17.0) | 0.6 |
| MSPSS: Family | 440 | 17.00 (15.00, 19.00) | 17.00 (15.00, 19.00) | 0.3 |
| MSPSS: Friends | 440 | 15.0 (11.0, 17.0) | 14.0 (12.0, 17.0) | >0.9 |

- Univariable analyses suggest associations between missingness and self-efficacy, positive reorientation and rumination/negative thinking at the p<0.2 level.
