# Supplementary material for: Sports-based mental health promotion for adolescents in rural Nepal: A pilot cluster-randomised controlled trial
Source: PLOS Glob Public Health. 2026 May 18;6(5):e0005991. doi: 10.1371/journal.pgph.0005991 (PMC13183228; doi:10.1371/journal.pgph.0005991)
Supplement: S7 Table — (DOCX) [file pgph.0005991.s008.docx]

**S7 Table: Multivariable analysis of predictors of missing data at endline (n=440)**

| **Predictors** | **Odds Ratios** | **95% CI** | **p** |
| --- | --- | --- | --- |
| Age | 1.64 | 1.44,  1.87 | **<0.001** |
| Cohabits with mother | 0.65 | 0.38,  1.13 | 0.124 |
| Currently studying | 0.69 | 0.26,  1.76 | 0.449 |
| Plays sport | 1.05 | 0.64,  1.74 | 0.847 |
| Dances | 0.70 | 0.41,  1.18 | 0.192 |
| Self-efficacy | 1.03 | 0.97,  1.10 | 0.302 |
| AERSQ: Positive reorientation | 1.01 | 0.91,  1.12 | 0.857 |
| AERSQ: Rumination / negative thinking | 1.07 | 1.00,  1.14 | 0.069 |

- We included variables in a multivariable analysis of predictors of missingness if significant in univariable analyses at p<0.2 level (S5 and S6 Tables). Only age was significant in multivariable analysis at p<0.05 level and was consequently included in the primary analysis model.
