# Supplementary material for: Sports-based mental health promotion for adolescents in rural Nepal: A pilot cluster-randomised controlled trial
Source: PLOS Glob Public Health. 2026 May 18;6(5):e0005991. doi: 10.1371/journal.pgph.0005991 (PMC13183228; doi:10.1371/journal.pgph.0005991)
Supplement: S8 Table — (DOCX) [file pgph.0005991.s009.docx]

**S8 Table: Moderation of effect size by gender**

| **Outcome** | **Difference in means^1^** | **Standard error^2^** | **95% CI^3^** |
| --- | --- | --- | --- |
| WEMWBS-14 | -1.87 | 1.46 | -4.59, 1.10 |
| WEMWBS-7 | -0.43 | 0.72 | -1.80, 0.97 |
| Functional impairment | 0.08 | 0.57 | -1.06, 1.20 |
| Depression (PHQ-A) | -1.41 | 0.73 | -2.87, -0.00 |
| Anxiety (GAD-7) | -0.5 | 0.72 | -1.82, 0.92 |
| Self-esteem | 0.53 | 0.63 | -0.69, 1.87 |
| Self-efficacy | -0.46 | 0.86 | -2.07, 1.18 |
| AERSQ: Positive reorientation | -0.15 | 0.58 | -1.38, 0.96 |
| AERSQ: Rumination/negative thinking | 0.09 | 0.59 | -1.08, 1.14 |
| AERSQ: Social support | 0.35 | 0.64 | -0.96, 1.62 |
| AERSQ: Aggressive outlet | -0.23 | 0.54 | -1.30, 0.82 |
| AERSQ: Creativity expression | 1.2 | 0.5 | 0.23, 2.16 |
| AERSQ: Distraction | 0.24 | 0.43 | -0.62, 1.11 |
| MSPSS: Total | -0.31 | 1.37 | -2.93, 2.33 |
| MSPSS: Significant others | -0.25 | 0.62 | -1.36, 1.02 |
| MSPSS: Family | -0.11 | 0.51 | -1.13, 0.80 |
| MSPSS: Friends | 0.06 | 0.58 | -1.12, 1.18 |

- S8 Table presents analyses to examine moderation of effect size by gender. If the difference in means was two or more times greater than the standard error we considered this as potentially important.
