# Supplementary material for: Sports-based mental health promotion for adolescents in rural Nepal: A pilot cluster-randomised controlled trial
Source: PLOS Glob Public Health. 2026 May 18;6(5):e0005991. doi: 10.1371/journal.pgph.0005991 (PMC13183228; doi:10.1371/journal.pgph.0005991)
Supplement: S10 Table — (DOCX) [file pgph.0005991.s011.docx]

**S10 Table: Cost of implementation and set up of SMART**

| **Total cost of the programme and each component** | **Total cost** (2024 US$) | **Share of cost** | **Average annual cost** (averaged over duration of programme component) |
| --- | --- | --- | --- |
| All components | 71,497 | 100% | 30,642 |
| Coaching session facilitation | 28,697 | 40% | 12,299 |
| Training and recruitment of sports coaches/staff | 10,556 | 15% | 4,524 |
| Melas | 16,984 | 24% | 7,279 |
| Development/adaptation | 4,733 | 7% | 2,028 |
| Monitoring & evaluation | 9,554 | 13% | 4,095 |
| Process evaluation | 974 | 1% | 417 |

- The combined economic cost of intervention set-up and implementation over the entire project duration.
