# Supplementary material for: Sports-based mental health promotion for adolescents in rural Nepal: A pilot cluster-randomised controlled trial
Source: PLOS Glob Public Health. 2026 May 18;6(5):e0005991. doi: 10.1371/journal.pgph.0005991 (PMC13183228; doi:10.1371/journal.pgph.0005991)
Supplement: S1 Text — (PDF) [file pgph.0005991.s012.pdf]

Full Application Form

Filter Questions

1 Is your study considered research as defined in the guidance icon information?

- (x) Yes
- ( ) No

2 Does your study require external ethical review by either the Health Research Authority (which includes the NHS REC and Social Care REC) or the Ministry of Defence REC?
See guidance icon for further information on the HRA and MOD REC ethical review remit.

- ( ) Yes
- (x) No

Data Collection

3 Select one category from the list below (categories are defined in the guidance icon).

My study involves:

- (x) a) Only primary data collection involving human subjects.
- ( ) b) Only analysis of pre-existing human subject data which is not in the public domain and contains identifiable personal data (see guidance icon for definition)
- ( ) c) Both primary data collection involving human subjects and analysis of pre-existing human subject data which is not in the public domain and contains identifiable personal data (see guidance icon for definition)
- ( ) d) Data collection not involving any of the above but presenting sensitive issues
- ( ) e) None of the above

4 Risk Checklist: Please indicate if your study involves any of the following risks:

- [x] a) The research involve participants who are vulnerable or unable to give informed consent or in a dependent position.
- [ ] b) Participants will take part in the study without their consent or knowledge at the time of participation or deception of some kind will be involved.
- [ ] c) The research topic may lead participants to disclose their involvement in activities that are illegal, could make them the target of personal or professional reprisals, or otherwise represent a threat to themselves or others.
- [ ] d) The study may induce psychological stress or anxiety, or produce humiliation or cause harm or negative consequences beyond the risks encountered in a participant's usual everyday life.
- [x] e) Participation in this research may identify urgent mental health risks, including, but not limited to, suicidal ideation and/or self-harm intent.
- [ ] f) There is a foreseeable likelihood that a participant's capacity to give fully informed consent may diminish throughout the course of the project i.e. early stage dementia, brain injury etc.
- [ ] g) The study involves imaging techniques such as MRI scans or ultrasound.
- [ ] h) The study involves sources of non-ionising radiation (e.g. lasers)
- [ ] i) The study involve physically invasive procedures or the collection of bodily materials (including collection of human tissue for purposes such as DNA/RNA analysis)
- [ ] None of the above.

5 Does the study involve the recruitment of participants under the age of 16?

- (●) Yes
- ( ) No

5(a) Will 'opt in' informed consent be obtained from the parents/guardians of all participants under the age of 16?

- (●) Yes
- ( ) No

Based on your answers to the above filter questions your research has been categorised as High Risk

You can now access an overview of the available sections of the application by selecting the navigate tile in the action panel on the left. Alternatively you can proceed through each section of the application by selecting the next tile.

Upon submission will be subject to review at the next relevant Research Ethics Subcommittee meeting. Meeting dates and submission deadlines can be found here

Section A: General Information

A Applicant Details

Title	First Name	Surname
Dr	Kelly	Rose-Clarke

Department: Global Health & Social Medicine

KCL Email: kelly.rose-clarke@kcl.ac.uk

A2 Applicant Status

Staff

A3 Applicant Role

Other

A4 What is your role in the project?

Co-Principal Investigator

A5 Is King's College London the research sponsor?

(•) Yes ( ) No

A6 Who is the Principal Investigator?

Title	First Name	Surname
Dr	Kelly	Rose-Clarke

Organisation: KCL

Email: kelly.rose-clarke@kcl.ac.uk

A7 Faculty/Institute/School

Please refer to the information icon if you are unsure of your Faculty/Institute/School.

Social Science and Public Policy

A9 Job Title

Lecturer in Global Mental Health

A13 Are there any other investigators/collaborators involved in the study?

- (•) Yes
- ( ) No

Co-Investigator/ Collaborator Details

Title	First Name	Surname
Dr	Nagendra	Luitel

Organisation: Transcultural Psychosocial Organization Nepal

Email: luitelnp@gmail.com

What is the role of this investigator?

Co-Principal Investigator

Co-Investigator/ Collaborator Details

Title	First Name	Surname
Dr	Joanna	Morrison

Organisation: University College London

Email: joanna.morrison@ucl.ac.uk

What is the role of this investigator?

Co-investigator

Co-Investigator/ Collaborator Details

Title	First Name	Surname
Mr	Nabin	Lamichhane

Organisation: CARE US

Email: nabinlc@hotmail.com

What is the role of this investigator?

Co-investigator

Co-Investigator/ Collaborator Details

Title	First Name	Surname
Dr	Jaya	Regmee

Organisation: Kathmandu Medical College

Email: jayaregmee@gmail.com

What is the role of this investigator?

Co-investigator

Section B: Project Information

B1 Project Title
A working title that accurately reflect the aims of the project.

Developing a sports-based mental health promotion intervention for adolescents in Nepal: a community engagement approach

B2 Anticipated start date for the collection of data: 17/04/2022

B3 Expected completion date of the project: 31/01/2024

B4 Is this a funded project?

- (●) Yes
- ( ) No

B4a How is the project being funded?

Externally funded

B4b Who is funding the project?

UKRI

B4c Have you been provided with a Funder Reference Number?

If you are unaware of your Grant Award Reference or Contract's Funder Reference please contact your Award Management Division campus team leader who will provide the relevant information.

- (selected) Yes
- No

B4c i) Please state your Funder Reference Number:

MR/T040181/1

B5 Please provide a summary of your project aims and objectives written in lay language that will be easily understandable to non-academic readers and non-specialists in your field. This summary should ideally be prefaced by the research question you hope to investigate in relation to your project, hypotheses to be tested, as well as a brief explanation of the academic background to the study.

Please Note: Applications to the Health Faculties RESC should include a full list of references/citations to back up the academic/scientific justification of the project.

Each year, one in five adolescents aged 10-19 experiences a mental disorder like depression or anxiety, and the rate is rising (Mokdad et al, 2016). We work in Nepal, a low-income country where there is a large population of adolescents at risk of mental disorders, but a lack of mental health care. An intervention is needed that can protect adolescents from mental disorders, is accessible to all adolescents, and is cheap and easy to sustain. One such intervention is mental health promotion, which focuses on improving positive behaviours and characteristics that protect mental health.

The study will be conducted in partnership with Transcultural Psychosocial Organization (TPO) Nepal. Our research aims to develop a mental health promotion intervention that uses sports groups to engage and improve the mental health of adolescents in Bardiya district, Nepal. We decided to focus on sports because there is evidence that sports activities improve mental health, adolescent participation in sports is supported by national and global adolescent policy, and adolescents across Nepal are already playing sport through a national network of sports clubs (Lubans et al, 2016).

The work is divided into three Work Packages (WPs).

WP1: Developing the intervention
To develop the intervention we will synthesise information from: (i) a review of the global and local literature on relevant sports-based interventions; (ii) semi-structured interviews and focus group discussions (FGDs) with adolescents, parents/caregivers, teachers, health workers, sports coaches, and other stakeholders with experience working with adolescents in the community; (iii) and transect walks where adolescents will take researchers on foot to places that adolescents like to go in their community, and discuss community norms in relation to adolescents' use of sports resources and facilities (Bond et al, 2019). We will recruit adolescent and adult community advisory groups to feedback on our findings and guide intervention development.

WP2: Translating and adapting tools
In parallel with WP1 we will translate and adapt tools to assess adolescent mental health and wellbeing. This will include FGDs and cognitive interviews with adolescents to assess the comprehensibility, acceptability, relevance, and completeness of tools.

WP3: Household validation/baseline survey
We will conduct a household survey of adolescents in four areas of Bardiya district which will serve two purposes: (i) it will enable us to validate the tools translated and adapted in WP2; (ii) the survey data will be used in a two-armed cluster randomised pilot trial to assess the feasibility of the intervention developed in WP1. The four areas will serve as four clusters. After the household survey, clusters will be randomised in a 1:1 ratio to control and intervention arms. The intervention will be implemented in intervention clusters for 10 months after which we will conduct a second household survey in all four clusters. Data from the first (baseline) and second (endline) household surveys will be used to explore differences between trial arms across outcomes for adolescent wellbeing and mental health. We will seek separate ethical approval for implementation of the intervention and the second household survey once the intervention has been finalised.

Our main research outcome will be a sports-based mental health promotion intervention that can be tested in a full scale trial. Our research will contribute important knowledge on sports-based mental health promotion that can be used by governments, researchers and organisations working in other low- and middle-income countries.

References
Mokdad AH, Forouzanfar MH, et al. Global burden of diseases, injuries, and risk factors for young people's health during 1990–2013: a systematic analysis for the Global Burden of Disease Study 2013. Lancet. 2016;387(10036):2383-401.
Lubans D, Richards J, et al. Physical activity for cognitive and mental health in youth: a systematic review of mechanisms. Pediatrics. 2016;138(3): e20161642.
Bond V, Ngwenya F, et al. Value and Limitations of Broad Brush Surveys Used in Community-Randomized Trials in Southern Africa. Qualitative Health Research. 2019;29(5):700-718.

B6 Where will the research be conducted? i.e in a facility within the college, in a private organisation, in a public place etc

Research will be conducted in the community, i.e. in schools, adolescents' homes, health services and sports facilities.

B7 If outside of the UK, please state the country/countries in which data collection is expected to occur.

Bardiya District, Nepal

B8 Selection of methodology from list: (select each that applies)

- [x] Questionnaires
- [x] Semi-structured interviews
- [ ] Unstructured Interviews
- [x] Focus Groups
- [ ] Observation
- [ ] Clinical Procedures or Interventions
- [ ] Non-clinical Procedures or Interventions
- [ ] Randomised Controlled Trial
- [ ] Oral history
- [ ] Analysis of pre-existing data from human participants
- [x] Audio/video recording or photography in a public place
- [x] Audio/video recording or photography in a private place
- [ ] Administration of substances (including food)
- [ ] Behavioural/Cognitive Testing
- [x] Other

Please note: If you intend to audio/ video record participants, these recordings are considered identifiable personal data under UK GDPR and therefore must be highlighted in Section E, as well as the relevant recruitment documents.

If other please specify:

Transect walks

If you are using any standardised methods for any of the above selected methodologies, please provide an overview of any standardised documentation to be used. Please provide full names and references where appropriate.
Please note you are not required to submit any standardised forms as supporting documents.

Questionnaires will include tools to assess mental health and wellbeing:
• Warwick Edinburgh Mental Wellbeing Scale - Tennant, R., Hiller, L., Fishwick, R. et al. The Warwick-Edinburgh Mental Well-being Scale (WEMWBS): development and UK validation. Health Qual Life Outcomes 5, 63 (2007). https://doi.org/10.1186/1477-7525-5-63
• Generalized Self-Efficacy Scale. Schwarzer, R., & Jerusalem, M. (1995). Generalized Self-Efficacy scale. In J. Weinman, S. Wright, & M. Johnston, Measures in health psychology: A user's portfolio. Causal and control beliefs (pp. 35-37). Windsor, UK: NFER-NELSON.
• Rosenberg Self Esteem Scale. Rosenberg, M. (1965). Society and the adolescent self-image. Princeton, NJ: Princeton University Press.
• Emotion Regulation Questionnaire for Children and Adolescents. Gullone E, Taffe J. The Emotion Regulation Questionnaire for Children and Adolescents (ERQ–CA): a psychometric evaluation. Psychol Assess. 2012;24:409–417. doi:10.1037/a0025777
• Depression Self-Rating Scale (DSRS). P. Birleson. The validity of depressive disorder in childhood and the development of a self-rating scale - a research report. J. Child Psychol. Psychiatry Allied Discip., 22 (1981), pp. 73-88
• Generalised Anxiety Disorder Assessment (GAD-7). Spitzer RL, Kroenke K, Williams JB, et al; A brief measure for assessing generalized anxiety disorder: the GAD-7. Arch Intern Med. 2006 May 22;166(10):1092-7.
• Locally developed measure of functional impairment. JORDANS, M., KOMPROE, I. H., TOL, W., KOHRT, B. A., LUITEL, N. P., MACY, R. D. & DE JONG, J. T. 2010.
Evaluation of a classroom-based psychosocial intervention in conflict-affected Nepal: a cluster randomized controlled trial. The Journal of Child Psychology and Psychiatry, 51, 818-826.
• Resilience Scale. Wagnild, G. M. & Young, H. M. (1993). Development and Psychometric Evaluation of the Resilience Scale. Journal of Nursing Measurement, 1, 165-178.
• Strengths and Difficulties Questionnaire. Goodman R, Meltzer H, Bailey V (1998) The Strengths and Difficulties Questionnaire: a pilot study on the validity of the self-report version. Eur Child Adolesc Psychiatry 7:125–130

Audio/video recording or photography
We will audio record interviews and FGDs. These will be conducted in a private place except for transect walks where adolescents will walk and talk with researchers through their village, visiting sports facilities and other relevant sites.
We will obtain informed consent to take photos of the community advisory groups to use in dissemination materials.

B9 Provide an explanation in lay language outlining each methodology of the study, as identified in question B8.

Below we detail methods related to each component of the work packages.

WP1: DEVELOPING THE INTERVENTION (MONTHS 1 TO 9, ~JAN TO JUL 22)
1.1. Literature reviews - global and local evidence for sports-based interventions for adolescent mental health. We will only be reviewing documents available in the public domain.

1.2. Formative research with adolescents
Objectives
(a) Explore the barriers and facilitators for participation in sports among girls, boys, school going, out of school, and disabled adolescents
(b) Explore which sports adolescents like (and play) and why they like them
(c) Explore the pros and cons of delivery of a sports intervention by teachers, sports coaches, or social mobilisers
(d) Explore what worries adolescents and how they deal with these worries/stressors, and how this is different among girls, boys, school going, out of school, and disabled adolescents
(e) Describe current sports and mental health activities, explore how they work and the factors affecting their implementation and response.

To address these objectives, we will conduct eight FGDs with in-school adolescents, four SSIs with out of school adolescents and one SSI with an adolescent who has a physical disability. Each FGD/SSI will last around an hour and be conducted with girls and boys aged 12 to 19, who like and dislike sport. FGDs and SSIs will be conducted by two research assistants (one asking questions and one note-taking) in a private place where adolescents feel comfortable, i.e. in a room in school or in a meeting space in the village. FGD and SSI topic guides will directly relate to the objectives above and are uploaded with this application. Because some adolescents may feel uncomfortable participating in an SSI on their own, we will ask if they would prefer to be interviewed with a friend, sibling or caregiver, in which case we will also obtain consent for this person's participation.

We will conduct two transect walks with adolescents. A transect walk is a participatory research method that entails a group of

participants (4 or 5) and researchers walking between two points to intentionally cross or transect a community. The group will explore community resources for sports and wellbeing by observing, asking, listening, and looking as they walk. This is a particularly useful method for data collection with adolescents who may feel uncomfortable in a more formal FGD/interview setting. Adolescents will meet with the researchers at an accessible venue (e.g. school) for an initial meeting to orientate them to the objectives of the study and to ask them about places where adolescents go in their community to meet their friends, play sport, and hang out. The researchers will explain that we'd like them to show and tell us about these places. The group agrees on a convenient time/date and place to start, and on a route that lasts about an hour. Whilst walking the researchers will ask adolescents about how they spend their time and will try to build rapport by chatting with them. When the group arrives at a place of interest the researchers will show them how to use the audio recorder and ask them to informally interview each other about the place. In our experience adolescents find this activity fun and it puts them in control of the discussion so they feel comfortable. The researchers listen to their discussions and ask further questions if needed. Only the adolescents' informal interviews are recorded. Researchers make written and mental notes which are later analysed, and they can refer to the audio recordings.

1.3. Formative research with adults
(a) Describe how sports are implemented in practice – including how they fit within the health/social development sector, and how they are financed
(b) Explore the challenges at a system and community level about how to run regular sports activities, and how these have been addressed.
(c) Explore who would be best to run a sports intervention

We will address these objectives through five FGDs with: local sports coaches; mothers of older adolescents (16-19 year); mothers of younger adolescents (12-15); fathers of older adolescents; and fathers of younger adolescents. We will also conduct eight SSIs with a district level coach, two community mobilisers (adults who work to implement government and NGO social and development programmes in the local area), two NGO-workers from local organisations working with adolescents, one health worker, and two school-based sports teachers. Topic guides will include questions to address the objectives and are uploaded with the application. FGDs and SSIs will be conducted in a private place, e.g. in a room in a school, in the TPO Nepal local office, or at sports facilities. They will be facilitated by two researchers (one to ask questions and one to take notes), will last around an hour and will be audio-recorded. Recordings will then be transcribed and translated into English for analysis.

1.4. Community advisory groups
We will recruit advisory groups in May/Jun 22 and conduct meetings with them over the course of our project, including the intervention development phase (this ethics application, i.e. until Sep 22) and the implementation/evaluation phase (a subsequent application, Sep 22 to ~Aug 23). Details of all planned meetings are provided here. We will issue members of the group with one consent form and information sheet for the duration of the project unless advised otherwise by REC.

Adolescent advisory group
Objectives:
(a) Help us validate (member check) our findings from the formative phase
(b) Give feedback on our ideas to address the barriers to participation in sports
(c) Select from a choice of sports which sport we should use for our intervention and help us understand why they feel that sport is the most appropriate.
(d) Give us feedback on key elements of the intervention in the design phase
(e) Give us feedback during implementation of how they and their peers feel about the intervention, any challenges and suggestions
(f) Help us evaluate the intervention – how it worked/did not work, and what contributed to that.
(g) Give feedback on our theory of change (at the end of the project)

We will recruit six to eight adolescents aged 12-19, who participated in the formative research. The group will comprise in and out of school adolescents, with equal numbers of boys and girls. We will hold four meetings of approximately 2-3 hours with the group:
Meeting 1 - Discuss the aims of the group, expectations and ground rules.
Meeting 2 - Feedback research findings and intervention plans and discuss them.
Meeting 3 – Discuss intervention theory of change and how the implementation of the intervention is going.
Meeting 4 – Discuss our findings from the evaluation

Adult advisory groups (x2)
Objectives:
(a) Help us member check our findings from the formative phase
(b) Help us plan and implement community activities to promote the intervention and promote community support for the intervention
(c) Give us feedback on the intervention (and our research approach) while it is being implemented.
(d) Help us evaluate the intervention – how it worked/did not work, and what contributed to that.
(e) Give feedback on our theory of change (at the end of the project)

Each group will comprise 6-8 members and include males and females. The first group will involve parents/caregivers of adolescents. The second will involve teachers, primary health care workers, sports coaches, and community mobilisers. We will hold three 1-2 hour meetings with these groups:
Meeting 1 – Feedback research findings and intervention plans and discuss them
Meeting 2 – Discuss intervention theory of change and how implementation is going
Meeting 3 – Discuss evaluation findings

Meetings with the advisory groups (adolescent and adult groups) will be held in the local TPO Nepal office or in another easily accessible space within the community which is acceptable to the groups. During meetings researchers will take notes and audio-

record discussions. These will not be transcribed but will be used by researchers to refer to whilst making notes. With the group's written permission (included in the consent form) researchers will also take photographs of group activities.

WP2: TRANSLATING AND ADAPTING TOOLS (MONTHS 3 TO 9, ~MAR TO JUL 22)
2.1. Translation
Bilingual speakers at TPO Nepal will translate the following tools to measure wellbeing from English to Nepali: the Warwick Edinburgh Mental Wellbeing Scale, the Schwarzer General Self-Efficacy Scale, the Rosenberg Self-Esteem Scale, and the Emotion Regulation Questionnaire Child and Adolescent Version.

2.2. FGDs and cognitive interviews with adolescents
We will conduct eight focus groups (6-8 adolescents per group): two with older girls (16-19 years); two with older boys (16-19 years); two with younger girls (12-15 years); two with younger boys (12-15 years). Through these FGDs we aim to explore the comprehensibility (Is the translation understandable?), acceptability (Is this question acceptable to ask?), relevance (Is the question locally appropriate based on lifestyle and behaviour?), completeness (Is this question assessing impairment or improvement?) of each tool in 2.1. Topic guides will include questions on each of these areas and are uploaded with the application. Each focus group will focus on two of the four tools.

Through 28 cognitive interviews with adolescents aged 12-19, we will explore how adolescents interpret each question included in the tool, and the different response options. (See Cognitive Interview Guide uploaded with the application for more detail).

Researchers will conduct FGDs and cognitive interviews in a private room in schools. Discussions and interviews will last approximately one hour and be audio-recorded. They will not be formally transcribed or translated but findings will be summarised using a structured form, and these will be used to revise the tools. Tools will then be back-translated into English by a bilingual speaker blinded to the original instruments. The study team will then review the original and back-translated versions.

WP3: HOUSEHOLD VALIDATION/BASELINE SURVEY (MONTHS 10 & 11, ~ AUG – SEP 2022)
We will conduct a household survey of adolescents aged 12-19. Data from this survey will serve two purposes:
i. We will analyse data from the household survey to assess the validity of the tools translated and adapted in WP2.
ii. The survey data will be used in analyses conducted as part of our pilot trial. The trial will comprise a baseline survey (the household survey), a period of implementation of the intervention, and an endline survey. (We will seek ethical approval for implementation and the endline survey once the intervention has been finalised.) We will obtain informed consent from participants for their data to be used for the purpose of analysing tools, and for the trial; it will not be possible for participants' data to be used for the purpose of one but not the other. Adolescents' participation in the household/baseline survey is not a prerequisite for, nor does it oblige them to participate in the intervention activities or the endline survey.

We will conduct the household survey in four areas of ~1000 population (~160 adolescents aged 12-19) in Bardiya District. These areas were selected because: they have community sports facilities, they are accessible by road, and their populations are ethnically diverse (and hence findings are more likely to be generalisable to other areas of Nepal). Moreover, the future pilot trial will be a cluster randomised controlled trial; these four areas will serve as the clusters, randomised 1:1 to intervention and control arms. The areas are separated geographically which will mitigate risk of contamination in the control arm.

A researcher will visit each household in the clusters and ask if there are any adolescents living in the household. Where appropriate consent is obtained, the researcher will then interview eligible adolescents using a smartphone or tablet pre-programmed with the survey.
living in the study area. The survey will include the translated and adapted versions of the Warwick Edinburgh Mental Wellbeing Scale, the Schwarzer General Self-Efficacy Scale, the Rosenberg Self-Esteem Scale, and the Emotion Regulation Questionnaire Child and Adolescent Version. We will also include questions on depression (Depression Self Rating Scale), anxiety (Generalised anxiety disorder assessment), resilience (Resilience Scale), functional impairment, pro-social behaviour (Strengths and Difficulties questionnaire), sociodemographic characteristics of adolescents and their household, and information about participation and preferences regarding sport. A draft of the survey is uploaded with our application.

If the summary of your methodology would be supported by a flowchart please attach this here (an editable flowchart can be found via the link in the guidance icon)

Documents

Type	Document Name	File Name	Version Date	Version	Size
Other	Sports flowchart	Sports flowchart.pdf	24/02/2022	1.0	31.0 KB

B10 I confirm that the researcher who will be administering all tests and/or procedures is competent in the methods.

- (•) Yes
- ( ) No

Section C: Participants

C1 Detail your projected number of participants and provide justification for this sample size.
Please note: For projects involving mixed methods and/or multiple participant groups, you should provide an estimate of the number of participants taking part in each method.

WP1: DEVELOPING THE INTERVENTION
1.2. Formative research with adolescents
8 FGDs: 6-8 adolescents per group = max 64
5 SSIs
2 transect walks: 4-5 girls and 4-5 boys per walk = max 10
SUBTOTAL = 79

1.3. Formative research with adults
5 FGDs: 6-8 adults per group = max 40
8 SSIs
SUBTOTAL = 48

1.4. Community advisory groups
Adolescent advisory group: 6-8 members = max 8
Adult advisory group (x2): 6-8 members = max 16
SUBTOTAL = 24

WP2: TRANSLATING AND ADAPTING TOOLS
2.2. FGDs and cognitive interviews with adolescents
8 FGDs: 6-8 adolescents = max 64
28 Cognitive interviews
SUBTOTAL = 92

WP3: HOUSEHOLD VALIDATION/BASELINE SURVEY
Survey participants: 4 clusters of ~1000 population; adolescents aged 12-19 constitute ~16% of population therefore 160 per cluster therefore max = 640 participants

Justification
Advisory groups, FGDs and SSIs, samples sizes are based on: (i) number of participants needed to represent diversity of caste/ethic groups, ages (adolescents), genders; (ii) our own experience in previous projects; (iii) expectation that some participants will drop out or not show up at research activities; (iv) the need to keep the sample small enough to facilitate close working relationships and meaningful participation.
For the validation/baseline survey, a sample of 4 clusters (~ 800 adolescents) will enable us to have enough statistical power to test convergent and divergent validity and perform a factor analysis, test randomisation and survey procedures across a range of settings, and estimate the intra-cluster correlation coefficient.

C2a What are the Inclusion Criteria? Where appropriate explain how you will screen your participants. *(The selection criteria should be clearly defined for multiple participant groups)*

WP1: DEVELOPING THE INTERVENTION
1.2. Formative research with adolescents
We will purposively sample adolescents for the following 8 FGDs:
- Girls aged 12-15 who like sport and are in school
- Girls aged 16-19 who like sport and are in school
- Girls aged 12-15 who do not like sport and are in school
- Girls aged 16-19 who do not like sport and are in school
- Boys aged 12-15 who like sport and are in school
- Boys aged 16-19 who like sport and are in school
- Boys aged 12-15 who do not like sport and are in school
- Boys aged 16-19 who do not like sport and are in school

For SSIs we will purposively sample the following adolescents aged 12-19: two out of school girls; two out of school boys; and one adolescent who has a physical disability.

Across FGDs and SSIs we will sample adolescents to maximise caste/ethnic diversity.

Transect walks: Walk 1 – girls aged 14-16, in-school and out-of-school, residing locally; Walk 2 – boys aged 14-16, in-school and out-of-school, residing locally

1.3. Formative research with adults
We will purposively sample for FGDs and SSIs as follows.
5 FGDs:
- 4 FGDs with parents/caregivers: equal numbers of mothers and fathers (or other relevant caregivers, see Section H7 for definitions) of adolescents aged 12-19. We will purposively sample across caste/ethnic groups
- 1 FGD with sports coaches (must be actively coaching sports activities and have done so for 2 years minimum)
8 SSIs:
- District level coach
- 2x community mobilisers (adults who work to implement government and NGO social and development programmes in the local area)
- 2x NGO-workers from local organisations working with adolescents
- Health worker
- 2x school-based sports teachers

1.4. Community advisory groups
Adolescent advisory group: participated in earlier SSIs or FGDs (1.2), who are interested and can give the time. We will endeavour to include at least on adolescent with a physical disability. The group will be comprised of older adolescents aged 14-16, in and out of school, equal numbers of girls and boys.

Adult advisory groups: Group 1 – parents/caregivers of adolescents (not those on adolescent advisory board), mixed gender group, representing different professions and caste/ethnic groups; Group 2 - teachers (at least one male and one female), health workers (e.g. doctor or nurse) sports coaches, community mobilisers.

WP2: TRANSLATING AND ADAPTING TOOLS
2.2. FGDs and cognitive interviews with adolescents
FGDs and cognitive interviews: equal number of boys and girls purposively sampled to represent the full age range (10-19 years) and caste/ethnic groups.

WP3: HOUSEHOLD VALIDATION/BASELINE SURVEY
Survey participants: Any adolescent boy or girl aged 12-19 years, in or out of school, married or unmarried, who lives in the study clusters.

C2b What are the Exclusion Criteria? Where appropriate explain how you will screen your participants. *(The selection criteria should be clearly defined for multiple participant groups)*

1.2. Formative research with adolescents
Purposively sampled so no exclusion criteria

1.3. Formative research with adults
Purposively sampled so no exclusion criteria

1.4. Community advisory groups
Anyone who knows that they are planning to move away from the study area during the project or is likely to be unable to attend meetings due to other commitments.

2.2. FGDs and cognitive interviews with adolescents
Adolescents who participated in the FGDs will not be able to participate in the cognitive interviews.

3. Household validation/baseline survey
Adolescents who do not live in the study clusters (e.g. adolescents who live outside the clusters but are visiting friends/family in the study area at the time of the survey).

C3 What are the upper and lower age limits? Provide justification for these where appropriate.

Adolescents will be 12-19 years old in order to include all ages in the Nepali secondary school system.

C4 How will potential participants be identified and approached?

Please note: If different recruitment methods will be used for participant groups, each group should be separately addressed.

1.2. Formative research with adolescents
With permission from the school principal, we will give a briefing to classes of students who are around 12-15 years old and to students who are around 16-19 years old. Among these adolescents we will identify which adolescents like and dislike sports using the following methods:
(a) Person bingo – Give adolescents a list of questions about their interests (one of which is “Do you enjoy sports?”). Adolescents write answers to these questions then walk around the room finding people who have answered the question in the same way they have. For each question they need to identify at least three people who gave the same answer.
(b) The chair game - Put a chair in the middle of the room. Ask for a volunteer, or the class identify the adolescent who likes wai wai (instant noodles) the most. They sit in the chair. Other adolescents stand at a distance depending on how much they like wai wai. If they like it a lot they stand closer to the person in the chair. If they like it less, they stand further away. Replace wai wai with sport. Play again.

We will note the names of students who like sports or do not like sports based on these two methods and ask them if they are interested in participating in an FGD or transect walk. Those who are interested will be given a consent form to take home, as well as a short survey where they provide their gender, disability status and ethnic group, and say whether they do any sport. We will recruit adolescents who return the completed survey and consent form, using the sampling strategy detailed in C2a. This will include an adolescent who has indicated in the survey that they have a disability. If we do not identify disabled adolescents in schools, we will ask if students or teachers know any adolescents with a disability in their community. If they do, we will ask them to pass on our contact information and the recruitment documents.

To recruit out of school adolescents we will ask in-school adolescents if they have any out of school friends. We will also ask adult participants (1.3) if they know any out of school adolescents. We will ask adolescents/adults to pass on the recruitment documents. If out of school adolescents are interested in participating, they can contact the research team directly or through their in-school friends/adult participants.

1.3. Formative research with adults
Parents/caregivers – In schools where we have permission from the principal, we will go to a classroom where we have not already asked adolescents to participate in formative research. We will ask adolescents to get into groups of 2 or 3 friends that live near them. We will do the chair game as above, but with each group splitting up and standing nearest or furthest from the chair. We will select several groups where some adolescents like and some don’t like sports. We will ask these adolescents if they would like their parent/caregiver to participate in a discussion about a sports programme for adolescent wellbeing. For those adolescents who would like their parent to participate we will ask about the best time and way to contact their parent/caregiver. A researcher will then speak to the parent/caregiver to explain the study and see if they are interested in participating. If so, we will ask the adolescent to take the recruitment documents home to their parent/caregiver to review and sign. If we find it difficult to recruit enough parents/caregivers using this approach, we will use a snowballing technique, i.e. ask parents who have already agreed to participate if they could ask other parents they know if they would like to participate.

Sports coaches, community mobilisers, NGO workers, and health workers – Using a snowball sampling approach, we will ask adolescents and adults participating in the formative research if they know of any sports coaches/community mobilisers/etc we could talk to and how best to contact them. We will also enquire through our local contacts at the municipality health office.

District level coach - Through our local contacts including at the All Nepal Football Association (ANFA) and the municipality health office, we will ask if there is a district level coach who would be interested in talking to us, and how we could contact them.

School sports teachers – At the start of the research we will organise a school staff meeting where we can present an overview of the study and respond to any questions from teachers and other staff members. During this meeting we will describe all the activities that will take place in the school. We will say that we would also like to hear from teachers involved in sports and, if they are interested in participating they can take an information sheet and consent form. We will explain at the meeting and again when we take consent that there is no pressure on staff to participate and no consequences if they decide not to.

1.4. Community advisory groups
We will ask adolescents and adults who participated in the formative research (1.2) if they would be interested in joining a group. We will also recruit members of the adult group through our local contacts at the municipality health office and ANFA.

2.2. FGDs and cognitive interviews with adolescents
We will recruit adolescents in a school in the study area after obtaining permission from the school principal. We will communicate our sampling framework to school staff members who will suggest classrooms where we can brief adolescents about the study. Those who are interested in participating will be given the relevant information sheet and consent form.

3. Household validation/baseline survey
Research assistants will visit each household in the study clusters. They will ask to speak to an adult to explain our study and enquire about any eligible adolescents living in the household. We will seek the adult’s permission before talking with adolescents about the study.

C5 Do you have a current or prior relationship with any potential participants? (This includes professional and/or personal relationships)

- ◯ Yes, I do have a current or prior relationships with potential participants.
- ◉ No, I do not have any current or prior relationships with potential participants.

C6 Gatekeeper Permission: Will you require an individual or organisation to grant you permission to approach/ access your intended participants? This includes gatekeepers contacting participants on your behalf

- ◉ Yes, I will be using a gatekeeper to access potential participants
- ◯ No, I will not be using a gatekeeper to access potential participants

C6a Will the gatekeeper be in a position of influence or authority over the participants?

- ◉ Yes, the gatekeeper is in a position of influence or authority over participants
- ◯ No, the gatekeeper will not be in a position of influence or authority over participants

C6b Outline who the gatekeeper is and how they will be used to facilitate recruitment.
Please note: Participants must only be approached once appropriate gatekeeper permission has been obtained.

Teachers and some adolescents will be recruited through schools in which case we will first obtain permission from the school principal. We will ask the principal and staff to identify classes where adolescents might be eligible to participate in our research.

C6c Please outline how gatekeeper permission will be obtained

The project manager will identify local schools and arrange to meet with the school principal to discuss the project. The principal will be fully informed and given the opportunity to ask questions before they are asked for permission.

C6d Explain how you will mitigate any pressure to participate that may be felt by potential participants as a result of the gatekeepers position.

Concerning adolescents, during briefings researchers will emphasise that adolescents' grades and education will not be affected in any way by their decision about whether to participate in the research. With regards to teachers, researchers will explain at a school staff meeting (please see answer to C4), and again when they take consent, that there is no pressure to participate and no consequences if they decide not to.

C7 Please specify any incentives being offered and a justification for their use.

We will cover transportation costs incurred by adolescents and adults participating in the SSIs, FGDs, transect walks or cognitive interviews. They will also be offered refreshments at the start of the activity (juice and biscuits).

For advisory group members (adults and adolescents), at each meeting they will be given Rs 1000 (£6.28) to cover transportation, and a small gift such as a pen, school bag, or pen drive.

No incentives will be offered to adolescents participating in the household validation/baseline survey.

Section C: Informed Consent

C8 Will informed consent be sought from all participants? (•) Yes ( ) No

C8a How will informed consent be obtained for each data collection method/participant group? Who will take consent and how will it be recorded?
Note: Justification must be provided if you will not be providing all participants with an information sheet and gaining written consent

For all participants, a trained researcher will obtain informed written consent. TPO Nepal's 16 year's of experience conducting mental health research in Nepal has found that it is better to give just one information sheet and consent form (rather separate versions for parents/caregivers and adolescents) because adolescents are often more educated and more literate than their parents, and both groups find it less confusing to have one form.

1.2. Formative research with adolescents / 2.2. FGDs and cognitive interviews with adolescents
Adolescents interested in participating will be given a consent form and information sheet (in Nepali) by a researcher, which they can take home and read with their parent/caregivers. Adolescents and parent/caregivers will be able to ask researchers any questions they have about the study either in person or by phone (contact details on the information sheet). Adolescents who return signed consent forms to school, signed by their parent/caregivers and themselves will be able to participate in the study. The Nepal Health Research Council (NHRC) Ethics Committee requires caregiver consent and adolescent assent for all adolescents under the age of 18, which we will adhere to in this study.

1.3. Formative research with adults
Adults (i.e. parents/caregivers, sports coaches, community mobilisers, NGO workers, health workers, and teachers) participating in FGDs and SSIs will be given an information sheet, and the researcher will verbally describe the study and what would be expected of them if they decided to participate. Adults will also be able to ask the researcher any questions. Where possible adults will be given at least 24 hours to decide if they would like to participate but this may not always be possible (see Section C9).

1.4. Community advisory groups
After participants have participated in the formative work, we will ask them if they might be interested in joining our advisory group. If they are, we will give them an information sheet and discuss what would be involved if they decided to participate. We will obtain informed written consent for all members of the advisory groups (and from a parent/caregiver if they are aged 17 or under). We will use one consent form and information sheet for all the meetings, which will include permission to audio record discussions and take photos to share in our dissemination materials. These will be collected from group members before the first meeting.

For adolescents, we will ask them to take the information sheet and consent form home and discuss it with their parent/caregivers. Parent/caregivers will also be invited to discuss the study with researchers, and contact details will be provided in the information sheet. Adolescents will be asked to return the signed consent form to school.

3. Household validation/baseline survey
Researchers will explain the study to the parent/caregiver and adolescent. They will be given an opportunity to ask questions before providing written consent.

C9 How long will participants be given to decide if they wish to participate?
Please provide justification if participants will be given less than 24 hours

For the most part we anticipate it will be possible to give participants more than 24 hours to decide. Participants for whom it may not be feasible include: adolescents living in remote villages that are difficult for the team to access (for example, some villages may not be accessible by road and researchers may have to trek to reach them); participants who, because of travel or their schedules have limited availability. It is important to represent these participant groups in the study, to understand their needs and priorities for intervention. Therefore, if it is not possible to give participants at least 24 hours we will discuss with them arranging the interview/FGD for later on the same day; making sure they have plenty of time to ask questions about the study; giving them chance to discuss the study with others (e.g. a husband, an older sibling, colleague, etc); and emphasising that they are under no pressure to participate.

C10 Detail the process by which participants may withdraw from the research both during the research and after it has been completed. A final withdrawal date should also be provided, after which participants may no longer withdraw their data from the study.

We will explain and emphasise to participants that they have the right to withdraw from the study without providing a reason. In the information sheet, we will inform participants that they have the right to withdraw their data up until one month after their participation, however it is not possible to remove an individual contribution from a focus group discussion or transect walk, due to the difficulty in isolating individual contributions and the fact that doing so if this were possible would remove valuable context to others' contributions.

Section D: High Risk Research

D1a Risk Identified: The research involves participants who are particularly vulnerable or unable to give informed consent or in a dependent position.

i) Explain how the participants are vulnerable in the context of the research

Participants will include adolescents aged 12-19. The age of majority in Nepal is 18 therefore it is expected that we will obtain parent/caregiver consent for those aged 12-17.
We will include at least one adolescent with a physical disability.

ii) How will you mitigate any potential risks to participants that may arise as a result of their vulnerability?

We will mitigate risks to adolescents in several ways:
i. Providing opportunities for community members to feedback on the study proposal through community advisory groups and open consultations
ii. Obtaining parental/caregiver consent for adolescents aged 12-17.
iii. Obtaining permission for recruitment from school principals
iv. Asking adolescents if they would prefer to be interviewed with a friend, sibling or caregiver (ensuring we also have consent for the participation of the additional person)
v. Choosing a place to conduct the SSI/FGD that is acceptable to adolescents and their parents/caregivers (e.g. a room in school)
vi. Referring adolescents to primary health care services or a TPO psychosocial counsellor as needed.

D1e Risk Identified: Participation in this research may identify urgent mental health risks, including, but not limited to, suicidal ideation and/or self-harm intent:

i) Explain how urgent mental health concerns could be identified as part of this study.

The research focuses on mental health and wellbeing, and whilst the emphasis is on positive mental health promotion (e.g. self-efficacy, wellbeing, self-esteem) participants may disclose concerns about their own or other people's mental health problems. For example, in the pilot trial we will measure symptoms of depression and anxiety, which may identify adolescents with mental health problems and/or suicidal ideation.

D1e(iii) What steps will be taken to mitigate any potential risks in response to the identification of urgent mental health concerns?
Please review the guidance icon before completing your response.

Information sheets will outline the topic (mental health promotion) and, where applicable, mention that participants will be asked about mental health problems. Researchers will be trained to ask questions in a sensitive and culturally respectful way, and to conduct SSIs/FGDs/surveys in a private space where participants feel comfortable. Participants will be reminded that they can stop or pause if they are uncomfortable, and that they don't have to answer questions if for any reason they do not want to. If participants do become visibly distressed, researchers will ask if they would like to take a break or finish and will follow up with them individually. Participants with high scores on depression or anxiety measuring tools indicative of a mental health problem, will be signposted to a local primary health centre where we will train health workers to provide mental health care. Where this is not practical or appropriate, we will offer adolescents the opportunity to speak to a psychosocial counsellor employed by TPO Nepal. Where participants disclose suicidal ideation, the researcher will activate a standardised referral pathway (used in our previous projects with TPO Nepal, also approved by KCL REC) which involves alerting the participants' parents/caregivers and arranging an urgent appointment with the TPO Nepal psychosocial counsellor.

D2 If there are any additional risks or burdens to participants that have not been addressed above, please provide further details and explain how these risks will be mitigated:

Our Nepali/English research team has extensive experience interviewing and implementing mental health research in rural Nepali contexts. TPO Nepal has been working with the Nepal Health Research Council to conduct research of the highest ethical standards since 2005. Research assistants receive training in quantitative and qualitative data collection including consent procedures, how to maintain confidentiality and data protection.

COVID-19 - The pandemic continues to affect Nepal, though there are currently no restrictions in place. We will be alert and responsive to changes in Nepali and UK rules and guidance, specifically information and guidance from the Nepal Health Research Council. We will encourage members of the research team to observe social distancing and hand washing recommendations, wear a mask, and conduct research activities outdoors where appropriate.

Participant time commitment - FGDs, interviews (SSIs and cognitive interviews) and transect walks will last around an hour. Members of the adolescent advisory group will be asked to participate in four meetings of 2-3 hours over 24 months. Members of the adult advisory group will be asked to participate in three 1-2 hour meetings over 24 months. This information is provided in the participant information sheets.

D3 What are the potential benefits to the participant?

Communities in control and intervention arms will benefit from having health workers trained in mental health care by our team.

D4 Will participants be guaranteed complete anonymity in the final report and any further research output/s?

(●) Yes ( ) No

D4a Please explain how you will ensure participants remain completely anonymous in the final report or any other research output/s.

We will not use any other identifying information about participants in our research outputs. Personal names, names of schools, or location information will all be removed from quotes used to report qualitative findings. Quantitative data will be pseudonymised prior to analysis and reported findings will not identify any individual participants.

Section E

E1 Does the project involve the collection and/or use of personally identifiable information (as outlined in UK GDPR)?

Identifiable information is data that can be used to identify an individual, either directly (such as full name, address, Twitter handle, etc) or indirectly through the combination of several pieces of data. The most common examples are names, contact details, audio/ video recordings, usernames etc. However, data that has the potential to indirectly identify a participant should also be treated as identifiable.

Please see the guidance icon for more examples of when data should be considered identifiable or contact the Research Governance Office: rgo@kcl.ac.uk

Please indicate which of the following applies:

- (•) Yes, the project involves the collection and/or use of identifiable information
- ( ) No, I will not be collecting and/or accessing any identifiable information for this project.

***Before completing the following questions, please ensure you have read the KCL Research Data Management Guidelines* and guidance on the UK General Data Protection Regulation (UK GDPR)**

Section E (I): UK Data Protection Requirements

E2 Who is the Data Controller? Please see the guidance icon for a definition of a Data Controller

- ( ) King's College London
- ( ) An External Individual/ Institution
- (•) King's College London is a Joint Data Controller with an External Individual/ Institution

E2a Details of the external Data Controller

Name	Dr Nagendra Prasad Luitel
Institution	TPO Nepal
Email	luitelnp@gmail.com / npluitel@tponepal.org.np

E3 Please state which of the following categories of personal data (relating to research participants) will be collected, processed or stored ***at any stage*** of the research project? (please select all that apply):

- [x] Name and/or signature (this includes those recorded on consent forms)
- [x] Date of Birth/ Age
- [x] Contact Details (email address, phone number, etc)
- [x] Identification Number (participant number, NHS number, staff number)
- [x] Location Data (full address, postcode, IP address etc)
- [ ] Online Identifier (identifiers provided by devices or apps, cookies etc)
- [x] Identifiable Image or Recording (photographs, video recordings and audio recordings) including interview recordings
- [x] Biographical Data (includes gender, marital status, employment history/job, etc.)
- [ ] Other

E4 Will any of the following special categories of personal data (relating to research participants) be collected, processed or stored at any stage of the research project from this point forward? (please select all that apply):

- [x] Race and/or ethnic origin
- [ ] Political opinions
- [x] Religious or philosophical beliefs
- [ ] Trade Union Membership
- [ ] Processing of genetic data
- [ ] Biometric data for the purpose of uniquely identifying a natural person
- [x] Health data
- [ ] Sex life
- [ ] Sexual orientation
- [ ] Criminal convictions or offences
- [ ] None of the above

E5 The UK GDPR identifies research as a 'public task' and as such you are advised to use 'public task' as your lawful basis for processing personal data. As you will also be collecting special category data, you are also required to state a condition for processing this data. As a KCL researcher, you are advised to use the 'archiving, research and statistics' condition for processing special category data.

Please confirm you will be processing personal and special category data under the 'Public task' and 'Archiving, research and statistics' lawful bases

(•) Yes, I will be processing personal data under the 'Public task' and special category data under the 'archiving, research and statistics' condition for processing

( ) No, I will be processing data under an alternative lawful basis and/or condition for processing

Section E (II) Data Handling, Protection and Storage during data collection & analysis

E6 In which format/s will the personal data be stored in while data collection and analysis is ***ongoing***?

- [x] Electronic Format
- [x] Hard Copy

E6a Electronic format - select all that apply:

Please note, where possible a KCL storage option should be selected in addition to any external storage

- [ ] KCL network drive
- [ ] KCL SharePoint
- [ ] NHS Network Drive
- [ ] KCL OneDrive
- [ ] Rosalind
- [ ] External hard drive
- [ ] USB
- [ ] KCL laptop
- [ ] Personal laptop
- [x] Other

If other, please specify:

All data collection is being conducted in Nepal by TPO Nepal employees who have no employment affiliation with KCL. KCL's role is that there is a KCL co-PI (Dr Kelly Rose-Clarke) who will be receiving de-linked pseudoanonymised datasets which will be analysed at KCL and stored on KCL servers. Dr Joanna Morrison, a co-I at UCL) will also be receiving de-linked pseudoanonymised data which she will analyse.

TPO Nepal researchers will collect quantitative data from participants using mobile phones and/or tablets, owned by TPO Nepal, which will be password protected and encrypted where possible. They will unlink the identifiers from the dataset using unique participant IDs. The dataset containing identifiers will be stored on a password-protected file on TPO's secure central server.

TPO Nepal researchers will make audio recordings on voice recorders. Recordings will be uploaded to the TPO server (and deleted from the recorder) and deleted from the server once they have been transcribed or, where transcription is not required (e.g. transect walks, cognitive interviews), after detailed notes have been taken. Transcriptions and notes will be stored on the TPO Nepal server. Any paper data (e.g. hand-written notes) will be stored in a locked filing cabinet at the TPO Nepal office.

E6a i) Can you confirm that the storage device is encrypted and, where possible, password protected?

- ○ Yes
- ◉ No

If no, please explain how data security is ensured:

All personal data will be stored at TPO Nepal and not shared with KCL. Dr Rose-Clarke (PI) will only receive de-linked pseudoanonymised datasets, transferred using a secure file transfer and storage service.

Across their various research projects, TPO Nepal ensure data security by storing datasets on their internal server, and using password protected devices (phone/tablet) to collect data. Their standards of data security meet and exceed data governance and ethical requirements in Nepal.

E6b Hard copy - select all that apply:

- ☐ Stored securely within the College
- ☑ Secure repository when in the field
- ☐ Stored securely on NHS premises
- ☐ Other

Please provide details of the specific location:

Paper copies of consent forms, SSI/FGD notes, etc will be kept in a locked cabinet in the TPO office.

E7 Data Access: **During data collection & analysis**, will data be shared with any researcher or individual outside of the immediate research team? (*Please note this includes sharing any audio/ video recordings with transcription services)*

- ○ No, data will not be shared with any other researcher or individual outside of the immediate research team during data collection and analysis
- ◉ Yes, data will be shared with another researcher or individual outside of the immediate research team during data collection and analysis (for example, for the purposes of transcribing the data)

E7a Select which of the following third parties data will be shared with **during data collection and analysis**:

- [ ] Third party transcription service
- [ ] Other third party private organisation (includes commercial companies)
- [x] Third party public organisation (includes universities)
- [ ] Third party charity organisation
- [ ] NHS
- [ ] Funder
- [ ] Other

E7a i) Please outline who will have access and why this is necessary

Co-I, Dr Joanna Morrison at UCL, will be working on qualitative data analyses and will receive pseudoanomysed de-linked data. There will not be any third parties receiving personal data.

E7b Will data be shared outside of the UK?

(•) Yes

( ) No

E7b i) Please state who data will the shared with and the country in which they are located

Data will be collected in Nepal and pseudoanonymised unlinked datasets will be shared with members of the research team in the UK.

E7c Will data be shared in an identifiable format? (*Please note this includes any audio/ video recordings)*

( ) Yes

(•) No

Please note: Data (either in an identifiable or anonymous format) should not be shared with external third parties unless there is an appropriate agreement in place. For guidance please contact the Contracts Team.

E8 Once **data analysis is complete** how will research data (including any participant contact details) be stored:

( ) Data will be stored in an identifiable format after analysis

(•) Data will be pseudonymised after analysis

( ) Data will be fully anonymised immediately after analysis

E8a Please outline how you will pseudonymise each category of personal data as selected under E3 & E4.

Data will be unlinked and identifying information will be securely retained by TPO Nepal.

Section E (III) Data Handling, Protection and Storage on completion of the research

E9 In which format/s will the personal data be stored following ***completion*** of data collection and analysis?

- [x] Electronic Format
- [ ] Hard Copy

E9a Electronic format - select all that apply:

Please note, where possible a KCL storage option should be selected in addition to any external storage

- [ ] KCL network drive
- [x] KCL SharePoint
- [ ] NHS Network Drive
- [ ] KCL OneDrive
- [ ] Rosalind
- [ ] External hard drive
- [ ] USB
- [ ] KCL laptop
- [ ] Personal laptop
- [x] Other

If other, please specify:

Pseudoanonymised unlinked data will be stored on TPO Nepal's password protected secure server.

E9a i) Can you confirm that the storage device is encrypted and, where possible, password protected?

- ( ) Yes
- (•) No

E9a ii) If no, please explain how data security is ensured:

The server is controlled by TPO Nepal who ensure data security through password-protected accounts for individual employees.

E10 Expected date that the data (including any participant contact details) will no longer be stored in an identifiable/pseudonymised format:

Please note: Data should only be stored in an identifiable format for as long as is absolutely necessary.

01/01/2025

E10a Please provide a justification to why personal data will be stored for the length of time indicated:

Beyond the study it is possible that some participants will continue to be supported by TPO Nepal (for example if they are referred to the psychosocial counsellor) in which case personal information will be retained to facilitate this.

E11 Data Retention Schedule

Research data should be stored in line with the KCL Data Retention Schedule. Please note that raw data should be stored in an ***anonymous/ pseudonymous*** format where possible.

- [x] I confirm that research data will be stored in line with the KCL Data Retention Schedule

E12 Data Access: Please confirm that no other researcher or individual outside of the immediate research team will have access to any personal data on completion of data collection and analysis

- (•) Yes, I confirm that no other researcher or individual outside of the immediate research team will have access to any personal data on completion of data collection and analysis
- ( ) No, another researcher or individual outside of the immediate research team will have access to personal data on completion of data collection and analysis

Section E (IV): Publication & Data Sharing on completion of the research

E13 Will any data from which participants could be identified be published (this could be direct quotes or biographical data that could lead to the identification of an individual)?

- ( ) Yes
- (•) No

E14 Will research data be shared with any external third parties **after data analysis is complete**?

- (•) Yes
- ( ) No

E14a Select which of the following third parties data will be shared with:

- [ ] External Project Supervisor
- [ ] Third party private organisation (includes commercial companies)
- [ ] Third party public organisation (includes universities)
- [ ] Third party charity organisation
- [ ] NHS
- [ ] Funder
- [x] Other

If other, please specify:

Other researchers who wish to analyse the data may request access. Requests will be reviewed on an individual basis by the Principal Investigators. Data information sheets will inform participants that their anonymised data may be shared for this purpose.

Please note: Data (either in an identifiable or anonymous format) should not be shared with external third parties unless there is an appropriate agreement in place. For guidance please contact the Contracts Team.

E14b Will data be shared in an identifiable format? (*Please note this includes any audio/ video recordings)*

- ( ) Yes
- (•) No

E15 Will data be archived for further use?

- (•) Yes
- ( ) No

E15a Will the archived data contain identifiable information?

- ( ) Yes
- (•) No

E16 Research Dissemination: How will results be disseminated?

- [ ] Internal report (thesis)
- [x] Journals
- [x] Conference
- [x] Other

If other please specify:

Dissemination workshops, policy briefs, website summaries, community meetings.

Section H: Insurance, Risks and Ethical Issues

H1 Does the project involve any of the Risk Assessment criteria outlined in the information icon guidance? (•) Yes ( ) No

H1a I confirm that I will complete a Risk Assessment Form which will be signed by my Supervisor or Head of Department prior to commencing data collection (•) Yes ( ) No

Please note: Your department should be able to provide you with a Risk Assessment Form. If they are unable to do so, please contact Health and Safety Services for further advice.

H2 Project Insurance Cover - Please indicate if your project involves any of the following -

- [ ] An overseas clinical trial
- [ ] Recruitment of overseas healthcare patients
- [ ] A physical or mental health intervention involving human subjects (see guidance icon for definition)
- [x] None of the above

Please note: *As your study does not involve any of the above, we can confirm that your study would be covered under the*

College's current insurance policies, subject to the relevant policy terms and conditions.

Please note that should there be any change to your study which changes your answer to question H2 above, then please contact Tania Pattenden (tel: 0207 848 3281 or e-mail: tania.pattenden@kcl.ac.uk) so that we can ensure appropriate insurance cover can be placed via consultation with our brokers. You will also need to submit a REMAS modification request to update your ethics protocol.

H3 Travel Insurance for overseas studies: I confirm that my travel insurance arrangements are as follows:

- (●) a) I will secure College travel insurance (see guidance icon for further details)
- ( ) b) I will secure personal travel insurance
- ( ) c) I do not require travel insurance as I will conduct the research in my country of legal residence
- ( ) d) I will not secure travel insurance for overseas travel

H4 I confirm that if Disclosure & Barring Service clearance is required for my study, this will be obtained prior to the commencement of data collection. (●) Yes ( ) No ( ) N/A

H6 Give the details of any other review body approvals or permissions obtained (including other Ethics Committees, peer review, R&D permission etc).

The proposal has been submitted for ethical approval to the Nepal Health and Research Council (http://nhrc.gov.np/). Permission to conduct the study will also be obtained from the Nepal Social Welfare Council (https://swc.org.np/) and local government.

H7 Give details of any other ethical issues which have not been addressed elsewhere in the application and explain how you will mitigate these risks.

Definition of parent/caregiver
In Nepal, many adolescents under 18 do not live with their biological parents due to e.g. migration or marriage. Consequently, their parents may be unavailable or unsuitable to provide consent for their son or daughter to participate in research. Based on consultation with local experts we clarify who can provide consent for the following adolescent groups:
- Adolescent under 18 living with his/her parents – Mother or father
- Married adolescent under 18 – Husband or wife (only if aged 18 or over), mother, father, mother-in-law, father-in-law.
- Adolescent under 18 not living with their parents: Adult appointed as the adolescent's caregiver (e.g. sibling aged 18+, aunt, uncle, grandmother, grandfather)
- Adolescent under 18 staying away from home for study or work – Mother, father, hostel warden
- Orphaned adolescent – Adult responsible for adolescent's care (e.g. aunt, uncle, grandmother, grandfather, sibling aged 18+), orphanage director

Developing appropriate, relevant recruitment documents for participants in Nepal
In my previous project in Nepal with TPO Nepal, approved by KCL REC (HR-18/19-8427), we discussed this issue with Annah Whyton, Research Ethics and Governance Manager until recently. Notably, KCL's information sheet and consent form templates are for the highest standard of GDPR compliance. Not all information sheets and consent forms necessarily have to meet this standard because the lawful basis for the research is a task in the public interest. Whilst there is a need to recognise GDPR, the focus on UK regulation is not helpful or necessarily relevant to participants in a Nepali setting. We decided that having more locally relevant documents was essential so that participants could fully understand the study and provide informed consent. We have therefore amended the KCL templates and will provide a more simplistic information sheet, as well as an additional data protection and storage information sheet available to participants on request, that outlines all the legal information on data protection and storage. We have removed the website link to KCL's use of personal data statement because many of our participants will not have access to the internet. This information has been included in the data protection and storage sheet instead. We will ensure that even if participants are not informed of all the GDPR aspects, the research team is acting in a way that is GDPR compliant in regards to data storage. Information sheets and consent forms uploaded with this application are based on the approved documents we used successfully in HR-18/19-8427 and are therefore tried and tested with adolescents and the community in Nepal. In line with advice from Clare Heard (RGO), we have edited information sheets to clarify that this is a KCL sponsored project and that KCL and TPO are joint data controllers. TPO Nepal have approved the participant-facing documents.

Contact with adolescents and children
The study team will have regular contact with adolescents. The Nepal ethics committee do not require researchers working with children to have the equivalent of a Disclosure and Barring Service (DBS) check, but TPO Nepal have their own robust training and reporting procedures in place. Dr Rose-Clarke underwent a DBS check in 2020 whilst at KCL and will liaise with HR to see if it needs updating.

Section I: Supporting Documents

I1 Participant Information Sheet

Information Sheet templates can be found under 'Recruitment documents'.

Documents

Type	Document Name	File Name	Version Date	Version	Size
Participant Information Sheet	SMART Information sheets and consent forms	SMART Information sheets and consent forms.docx	29/03/2022	3.0	228.5 KB

Consent form (if applicable)

I2 Consent form (if applicable)

Recruitment documents for parents/carers (if applicable)

I3 Information Sheet(s) and Consent Form(s) for parents-carers

Documents

Type	Document Name	File Name	Version Date	Version	Size
Participant Information Sheet	SMART Information sheets and consent forms	SMART Information sheets and consent forms.docx	29/03/2022	3.0	228.5 KB

Questionnaire/Survey template/s

I4 Questionnaire/Survey template/s

Documents

Type	Document Name	File Name	Version Date	Version	Size
Questionnaires	Warwick Edinburgh Mental Wellbeing Scale	WEMWBS.pdf	24/02/2022	1.0	29.3 KB
Questionnaires	Schwarzer General Self-Efficacy Scale	Self-efficacy.pdf	24/02/2022	1.0	175.8 KB
Questionnaires	Rosenberg Self Esteem Scale	Self_Measures_for_Self-Esteem_ROSENBERG_SELF-ESTEEM.pdf	24/02/2022	1.0	68.4 KB
Questionnaires	WP2.2 Emotion Regulation Questionnaire (CA version)	ERQ_CA.pdf	24/02/2022	1.0	125.9 KB
Questionnaires	WP3 Draft ado_validation,baseline survey	Example_ado_validation,baseline,endline survey.xlsx	25/02/2022	1.0	36.9 KB

List of Indicative questions or topic guides

I5 List of Indicative questions or topic guides

Documents

Type	Document Name	File Name	Version Date	Version	Size
Interview guide	WP1.2 FGD with adolescents v1	FGD with adolescentsv1.docx	24/02/2022	1.0	18.0 KB
Interview guide	WP1.2 Out of school SSIsv1	Out of school SSIsv1.docx	24/02/2022	1.0	17.9 KB
Interview guide	WP1.2 Transect walk	Transect walkv1.docx	24/02/2022	1.0	15.8 KB
Interview guide	WP1.2 SSI disabled adolescent	SSI disabled adolv1.docx	24/02/2022	1.0	18.4 KB
Interview guide	WP1.3 SSI comm mobiliser	SSI commobv1.docx	24/02/2022	1.0	16.9 KB
Interview guide	WP1.3 SSI sports teachers	SSI sports teachersv1.docx	24/02/2022	1.0	17.1 KB
Interview guide	WP1.3 FGD caregivers	FGD:Grp Ints caregiversv1.docx	24/02/2022	1.0	18.0 KB
Interview guide	WP1.3 SSI District level sports coach	SSI District level sports coach v1.docx	24/02/2022	1.0	17.7 KB
Interview guide	SSI sports coachesv1	SSI sports coachesv1.docx	24/02/2022	1.0	16.8 KB
Interview guide	WP2.2 TPO Nepal Instrument Transcultural Translation	TPO Nepal Instrument Transcultural Translation.docx	24/02/2022	1.0	23.9 KB
Interview guide	WP2.2 Cognitive Interviewing Guide	Cognitive Interviewing Guide.docx	24/02/2022	1.0	24.8 KB

Evidence of any other approvals or permissions (includes gatekeeper, R&D, other ethical approvals) (if applicable)

I6 Evidence of any other approvals or permissions (includes gatekeeper, R&D, other ethical approvals)

Approach letters to gatekeeper organisations (if applicable)

I7 Approach letters to gatekeeper organisations

Advertisement document (email, poster, flyer etc) (if applicable)

I8 Advertisement document (email, poster, flyer etc)

Cover Letter (for amendments and modifications) (if applicable)

I9 Cover Letter (for amendments and modifications)

Documents

Type	Document Name	File Name	Version Date	Version	Size
Other	Cover_letter_240222	Cover_letter_240222.docx	25/02/2022	1.0	95.1 KB
Other	Cover letter 230322	Cover letter 230322.docx	29/03/2022	1.0	84.1 KB

Other (if applicable)

I10 Other

Researcher/Applicant

J1 Researcher/Applicant Signature

I undertake to abide by accepted ethical principles and appropriate code(s) of practice in carrying out this study. The information supplied above is to the best of my knowledge accurate. I have read the Application Guidelines and clearly understand my obligations and the rights of participants, particularly as regards obtaining valid consent. I understand that I must not commence research with human participants until I have received full approval from the ethics committee.

Please note that in order to authorise your application you must sign off using your KCL email address i.e. joe.bloggs@kcl.ac.uk and your KCL password.

Signed: This form was signed by Kelly Rose-Clarke (kelly.rose-clarke@kcl.ac.uk) on 29/03/2022 11:49 AM
