# Supplementary material for: Sports-based mental health promotion for adolescents in rural Nepal: A pilot cluster-randomised controlled trial
Source: PLOS Glob Public Health. 2026 May 18;6(5):e0005991. doi: 10.1371/journal.pgph.0005991 (PMC13183228; doi:10.1371/journal.pgph.0005991)
Supplement: S2 Text — (PDF) [file pgph.0005991.s013.pdf]

Full Application Form

Filter Questions

1 Is your study considered research as defined in the guidance icon information? (x) Yes ( ) No

2 Does your study require external ethical review by either the Health Research Authority (which includes the NHS REC and Social Care REC) or the Ministry of Defence REC?
See guidance icon for further information on the HRA and MOD REC ethical review remit.

( ) Yes
(x) No

Data Collection

3 Select one category from the list below (categories are defined in the guidance icon).

My study involves:

(x) a) Only primary data collection involving human subjects.
( ) b) Use of identifiable human subject data that is not in the public domain. This includes analysis of previously collected data (including human tissue) and/or the use of existing data that has not previously been used for research purposes (such as data taken from private social media groups)
( ) c) Both primary data collection involving human subjects and the use of pre-existing human subject data as outlined above
( ) d) Data collection not involving any of the above but presenting sensitive issues
( ) e) None of the above

4 Risk Checklist: Please indicate if your study involves any of the following risks:

- [ ] a) The research involves participants who are vulnerable or unable to give informed consent or in a dependent position.
- [ ] b) Participants will take part in the study without their consent or knowledge at the time of participation or deception of some kind will be involved.
- [ ] c) The research topic may lead participants to disclose their involvement in activities that are illegal, could make them the target of personal or professional reprisals, or otherwise represent a threat to themselves or others.
- [ ] d) The study may induce psychological stress or anxiety, or produce humiliation or cause harm or negative consequences beyond the risks encountered in a participant's usual everyday life.
- [x] e) Participation in this research may identify urgent mental health risks, including, but not limited to, suicidal ideation and/or self-harm intent.
- [ ] f) There is a foreseeable likelihood that a participant's capacity to give fully informed consent may diminish throughout the course of the project i.e. early stage dementia, brain injury etc.
- [ ] g) The study involves imaging techniques such as MRI scans or ultrasound.
- [ ] h) The study involves sources of non-ionising radiation (e.g. lasers)
- [ ] i) The study involve physically invasive procedures or the collection of bodily materials (including collection of human tissue for purposes such as DNA/RNA analysis)
- [ ] None of the above.

Based on your answers to the above filter questions your research has been categorised as High Risk

You can now access an overview of the available sections of the application by selecting the navigate tile in the action panel on the left. Alternatively you can proceed through each section of the application by selecting the next tile.

Upon submission will be subject to review at the next relevant Research Ethics Subcommittee meeting. Meeting dates and submission deadlines can be found here

Section A: General Information

A Applicant Details

Title	First Name	Surname
Dr	Kelly	Rose-Clarke

Department: Global Health and Social Medicine

KCL Email: kelly.rose-clarke@kcl.ac.uk

A2 Applicant Status

Staff

A3 Applicant Role

Principal Investigator

A5 Is King's College London the research sponsor?

(•) Yes ( ) No

A7 Faculty/Institute/School

Please refer to the information icon if you are unsure of your Faculty/Institute/School.

Social Science and Public Policy

A9 Job Title

Senior Lecturer in Global Mental Health

A13 Are there any other investigators/collaborators involved in the study?

(•) Yes

( ) No

Co-Investigator/ Collaborator Details

Title	First Name	Surname
Mr	Nagendra	Luitel

Organisation: Transcultural Organization Nepal

Email: luitelnp@gmail.com

What is the role of this investigator?

Co-principal investigator

Co-Investigator/ Collaborator Details

Title	First Name	Surname
Dr	Joanna	Morrison

Organisation: UCL

Email: joanna.morrison@ucl.ac.uk

What is the role of this investigator?

Co-Investigator

Co-Investigator/ Collaborator Details

Title	First Name	Surname
Dr	Jaya	Regmee

Organisation: Kathmandu Medical College

Email: jayaregmee@gmail.com

What is the role of this investigator?

Co-investigator

Co-Investigator/ Collaborator Details

Title	First Name	Surname
Mr	Nabin	Lamichhane

Organisation: CARE US

Email: nabinlc@hotmail.com

What is the role of this investigator?

Co-investigator

Section B: Project Information

B1 Project Title

A working title that accurately reflect the aims of the project.

Piloting a sports-based mental health promotion intervention for adolescents in Nepal

B2 Anticipated start date for the collection of data: 01/11/2022

B3 Expected completion date of the project: 30/04/2024

B4 Is this a funded project?

- (•) Yes
- ( ) No

B4a How is the project being funded?

Externally funded

B4b Who is funding the project?

UKRI

B4c Have you been provided with a Funder Reference Number?

If you are unaware of your Grant Award Reference or Contract's Funder Reference please contact your Award Management Division campus team leader who will provide the relevant information.

- (•) Yes
- ( ) No

B4c i) Please state your Funder Reference Number:

MR/T040181/1

B5 Please provide a summary of your project aims and objectives written in lay language that will be easily understandable to non-academic readers and non-specialists in your field. This summary should ideally be prefaced by the research question you hope to investigate in relation to your project, hypotheses to be tested, as well as a brief explanation of the academic background to the study.

Please Note: Applications to the Health Faculties RESC should include a full list of references/citations to back up the academic/scientific justification of the project.

Research question: Is a sports-based intervention to promote adolescent mental health feasible and acceptable among adolescents in rural Nepal?

Each year, one in five adolescents aged 10-19 experiences a mental disorder like depression or anxiety, and the rate is rising (Mokdad et al, 2016). We are working in Nepal, a lower middle-income country where there is a large population of adolescents at risk of mental disorders, but a lack of mental health care. An intervention is needed that can protect adolescents from mental disorders, is accessible to all adolescents, and is cheap and easy to sustain. One such intervention is mental health promotion, which focuses on improving positive behaviours and characteristics that protect mental health (Barry et al, 2013).

This study is the second part of a project to develop and pilot a mental health promotion intervention that uses sports to engage and improve the mental health of adolescents in Bardiya district, Nepal. The first part of the project (formative work to develop the intervention and the baseline survey) received approval from KCL REC in April 2022 (HR/DP-21/22-27152, uploaded for reference). In this application, as advised by KCL REC for the second part of the project, we are seeking approval for: (i) implementation of the intervention; (ii) the endline survey; and (iii) the process evaluation. The Nepal Health Research Council has already provided ethical approval for the first and second parts of the project (see uploaded letter of approval).

Implementation of the intervention
The intervention aims to increase mental wellbeing, emotion regulation, self-efficacy, self-esteem, and social support, and decrease depression among adolescents in rural Nepal. It will do this by (1) engaging adolescents in sports coaching which includes activities to promote their life skills and values, (2) sports melas (festivals) involving sports matches, demonstrations and performances, (3) community theatre performances to raise local awareness about the intervention, adolescent mental health and the benefits of sports. Sports coaches and sports teachers will facilitate the sports coaching, assisted by assistant coaches who are young people aged 15-24 recruited locally. The coaching activities will be open to any adolescent though we will specifically target adolescents aged 12-19 living in the study clusters. Parents, siblings and other community members will be invited to the melas and community theatre performances.

Endline survey
The impact of the intervention will be evaluated at the community level through a parallel-group, two-arm, superiority, pilot cluster-randomised controlled trial with four clusters (two intervention and two control clusters). The control will be treatment as usual. The cluster unit will be a community of approximately 1000 population (~160 adolescents aged 12-19). The trial will involve two cross-sectional household surveys of adolescents, one at pre-intervention (baseline) and one post intervention (endline), in all four clusters. The main analysis will be a cross-sectional comparison of data from the endline survey, adjusted for baseline differences. We already have ethical approval for the baseline survey and this will start in September. The endline survey will be a repeat of the baseline survey with additional questions related to intervention participation.

Process evaluation
The process evaluation aims to capture information about how the intervention is being implemented, how acceptable it is, potential mechanisms behind its effects, and challenges experienced in the field. It will include a review of intervention documents, focus group discussions (FGDs) with adolescents who have and have not participated in intervention activities, observations of intervention activities and descriptive analyses of data on attendance, activities, and characteristics of participants.

Our main research outcome will be a sports-based mental health promotion intervention that can be tested in a future, full-scale randomised controlled trial. Our research will contribute important knowledge on sports-based mental health promotion that can be used by governments, researchers and organisations working in other low- and middle-income countries.

References
Mokdad AH, et al. Global burden of diseases, injuries, and risk factors for young people’s health during 1990–2013: a systematic analysis for the Global Burden of Disease Study 2013. Lancet. 2016;387(10036):2383-401.
Barry, Margaret M., et al. A systematic review of the effectiveness of mental health promotion interventions for young people in low and middle income countries. BMC public health 13.1 (2013): 1-19.

B6 Where will the research be conducted? i.e in a facility within the college, in a private organisation, in a public place etc

Research will be conducted in the community, i.e. in schools, adolescents' homes, and sports facilities.

B7 If outside of the UK, please state the country/countries in which data collection is expected to occur.

Nepal

B8 Selection of methodology from list: (select each that applies)

- [x] Questionnaires
- [x] Semi-structured interviews
- [ ] Unstructured Interviews
- [x] Focus Groups
- [x] Observation
- [ ] Clinical Procedures or Interventions
- [x] Non-clinical Procedures or Interventions
- [x] Randomised Controlled Trial
- [ ] Oral history
- [ ] Analysis of pre-existing data from human participants
- [ ] Audio/video recording or photography in a public place
- [ ] Audio/video recording or photography in a private place
- [ ] Administration of substances (including food)
- [ ] Behavioural/Cognitive Testing
- [ ] Other

Please note: If you intend to audio/ video record participants, these recordings are considered identifiable personal data under UK GDPR and therefore must be highlighted in Section E, as well as the relevant recruitment documents.

If you are using any standardised methods for any of the above selected methodologies, please provide an overview of any standardised documentation to be used. Please provide full names and references where appropriate.

Questionnaires in the endline survey will include all those used in the baseline survey:
• Warwick Edinburgh Mental Wellbeing Scale - Tennant, R., Hiller, L., Fishwick, R. et al. The Warwick-Edinburgh Mental Well-being Scale (WEMWBS): development and UK validation. Health Qual Life Outcomes 5, 63 (2007). https://doi.org/10.1186/1477-7525-5-63
• Generalized Self-Efficacy Scale. Schwarzer, R., & Jerusalem, M. (1995). Generalized Self-Efficacy scale. In J. Weinman, S. Wright, & M. Johnston, Measures in health psychology: A user's portfolio. Causal and control beliefs (pp. 35-37). Windsor, UK: NFER- NELSON.
• Rosenberg Self Esteem Scale. Rosenberg, M. (1965). Society and the adolescent self-image. Princeton, NJ: Princeton University Press.
• Adolescents' Emotion Regulation
Strategies Questionnaire. Zhou Y, Daukantaite˙ D, Lundh L-G, Wångby-Lundh M and Ryde A (2020)
Adolescents' Emotion Regulation
Strategies Questionnaire: Initial
Validation and Prospective
Associations With Nonsuicidal SelfInjury and Other Mental Health Problems in Adolescence and Young
Adulthood in a Swedish Youth Cohort. Front. Psychiatry 11:462.doi: 10.3389/fpsyt.2020.00462
• Depression Self-Rating Scale (DSRS). P. Birleson. The validity of depressive disorder in childhood and the development of a self-rating scale - a research report. J. Child Psychol. Psychiatry Allied Discip., 22 (1981), pp. 73-88
• Generalised Anxiety Disorder Assessment (GAD-7). Spitzer RL, Kroenke K, Williams JB, et al; A brief measure for assessing generalized anxiety disorder: the GAD-7. Arch Intern Med. 2006 May 22;166(10):1092-7.
• Locally developed measure of functional impairment. JORDANS, M., KOMPROE, I. H., TOL, W., KOHRT, B. A., LUITEL, N. P., MACY, R. D. & DE JONG, J. T. 2010.
Evaluation of a classroom-based psychosocial intervention in conflict-affected Nepal: a cluster randomized controlled trial. The Journal of Child Psychology and Psychiatry, 51, 818-826.

We will also include:
• Multidimensional Scale of Perceived Social Support. Zimet GD, Dahlem NW, Zimet SG, Farley GK. The Multidimensional Scale of Perceived Social Support. Journal of Personality Assessment 1988;52:30-41
• Support and Empowerment of Youth and Attachment to the Neighborhood subscales of the Adolescents' Developmental Assets in the Neighborhood Scale. Oliva Alfredo, Antolín Lucía, López Ana María. Development and validation of a scale for the measurement of adolescents' developmental assets in the neighborhood. Social Indicators Research. 2012;106(3):563–576.

Audio/video recording or photography
We will audio record interviews and FGDs. These will be conducted in a private place.

B9 Provide an explanation in lay language outlining each methodology of the study, as identified in question B8.

Below we detail methods related to each component for which we are seeking approval.

Implementation of the intervention (10 months, Dec/Jan 2022 to Oct 2023)
The intervention aims to improve adolescent mental wellbeing through sports coaching and adolescent clubs. It will be implemented in two of the four study clusters.

Sports coaching: Sports coaching: This will involve weekly sessions for around 90 min, facilitated by a sports coach, teacher, and assistant coaches. Sessions will take place in schools or community sports facilities before or after school or at weekends. Adolescents will be offered coaching in different sports such as football, dance and martial arts/self defence. Each session will involve an activity focused on promoting adolescents' mental wellbeing by building self esteem, self efficacy and skills including team building, communication, and interpersonal skills. The activities will include games, role-play, discussions, and relaxation exercises. The remainder of the session will focus on practicing sport through drills, training and friendly competitions/matches. Coaching will be open to any adolescent wishing to participate but will target adolescents aged 12-19 living in the study clusters. Sports coaches will engage with existing community groups (e.g. mothers' groups, micro-finance groups, parent-teacher committee) and parents to raise awareness about adolescent mental health, the benefits of sports, and the intervention activities.

Sports melas: With the help of adolescents, coaches will organise sports melas (festivals) at the start, midway and at the end of the 10-month implementation period. Melas will involve football matches, martial arts demonstrations, and dance performances. Adolescents will invite their family and other community members to watch.

Community theatre: A local community theatre group will put on scripted performances in the intervention clusters. Performances will aim to raise the community's awareness about the intervention activities, adolescent mental health needs and the benefits of sports. Performances will take place at the sports melas and other community meetings.

Adolescents can attend as many or as few of the intervention activities (coaching, melas, theatre performances) as they wish. They can take part in the intervention activities without agreeing to participate in the research (i.e. baseline and endline surveys and process evaluation).

Facilitator training: Sports coaches, teachers and assistant coaches will be trained by supervisors, and will conduct practice activities with smaller groups of adolescents.

In control and intervention clusters we will provide mental health training for local health workers including psychoeducation and information about services for referral. We will provide similar training for teachers but only in intervention clusters. We will distribute sports equipment at the municipality level. During the intervention implementation period adolescents will have access to any sports coaching/activities as usual.

Randomisation and allocation to the intervention: The study area is four communities of approximately 1000 population (~160 adolescents aged 12-19), two in Geruwa municipality and two in Madhuvan municipality in Bardiya District. Each community represents one cluster. The four communities were selected because: they are far enough apart and/or separated by natural boundaries (e.g. forest) so that it is unlikely that adolescents from the control communities will participate in activities in intervention communities and vice versa; they have a local secondary school and sports facilities; they are accessible to the study team by road; and their populations are ethnically diverse (and hence findings are more likely to be generalisable to other areas of Nepal). We will obtain permission from bolmunsa (community leaders) in these communities.

We will randomise communities before the baseline survey finishes in October 2022. This will be done by a statistician who does not know the study setting, and will be stratified by municipality – i.e. first the two clusters in Geruwa will be randomised 1:1 to intervention: control, then the two in Madhuvan. The statistician will use software to conduct the randomisation.

Endline survey (November and December 2023)
We will conduct a household survey of adolescents aged 12-19 in the four study clusters. The purpose of the survey is to assess any effects of the intervention. The endline survey will run in the same way as the baseline survey: a researcher will visit each household in the clusters and ask if there are any adolescents living there. After appropriate consent is obtained, the researcher will interview eligible adolescents using a smartphone or tablet pre-programmed with the survey. The survey will include translated/adapted versions of the tools listed in B8 (e.g. Warwick Edinburgh Mental Wellbeing Scale, Schwarzer General Self-Efficacy Scale, Rosenberg Self-Esteem Scale, and Adolescents' Emotion Regulation Strategies Questionnaire). We will also include questions on sociodemographic characteristics of adolescents and their household, and information about participation and preferences regarding sports and the intervention. We will conduct a cost analysis of the intervention so we will also include questions about costs related to participating in intervention activities such as transport costs and any lost earnings. A draft of the survey is uploaded with our application. The survey will take around 20 minutes to complete. Adolescents who participated in the baseline survey or intervention activities are under no obligation to participate in the endline survey. Similarly, adolescents can participate in the endline survey regardless of whether they participated in the baseline survey or intervention activities.

Process evaluation (conducted in parallel with intervention implementation and endline survey)
This aims to explore how the intervention is implemented, including fidelity, exposure and reach, mechanisms through which the intervention brings about change, and contextual factors that might affect outcomes and how the intervention is implemented. We will collect process evaluation data in intervention clusters only, at the midpoint of the intervention (midline) and at the end (endline). We will analyse quantitative data from the baseline and endline survey and conduct 24 focus group discussions and 2 interviews with key stakeholders (i.e. adolescents sports coaches, teachers, parents). Interviews and focus groups will last around one hour and be conducted in a private, quiet place where the participants feel comfortable, for example in schools (adolescents, teachers), a community building such as a health post or sports hall (sports coaches, parents, adolescents), the study team's local office (coaches, community mobilisers) or at the participant's home (adolescents, parents).

Adolescents will be recruited at intervention sessions (participants) or in schools (non-participants). Parents/caregivers will be recruited through coaches and teachers. Coaches, assistant coaches and teachers will be approached directly by the research team.

Adolescents will be purposively sampled based on their gender, age and participation (or not) in the intervention. Caregivers will be purposively sampled based on the gender of their child – we would like a mix of genders of adolescents. Teachers will be purposively sampled as the key point person in the school for the intervention. All coaches and assistant coaches will be sampled. Sampling will also be informed by any differences that we observe whilst implementing the intervention.

We will analyse transcripts from interviews and FGDs and observation notes in English in Nvivo using the framework method, informed by our theory of change. We will analyse quantitative data from reporting forms using Excel.

If the summary of your methodology would be supported by a flowchart please attach this here (an editable flowchart can be found via the link in the guidance icon)

Documents

Type	Document Name	File Name	Version Date	Version	Size
Other	Sports flowchart	Sports flowchart.pdf	22/09/2022	1.0	13.4 KB

B10 I confirm that the researcher who will be administering all tests and/or procedures is competent in the methods.

- (•) Yes
- ( ) No

B11 If applicable, please explain any context specific factors relating to your research that may be helpful to the committee when reviewing your application.

For example, an explanation of a novel research method in lay language or an overview of any cultural values or belief systems that have informed your process of obtaining informed consent.

Definition of parent/caregiver
In Nepal, many adolescents under 18 do not live with their biological parents due to e.g. migration or marriage. Consequently, their parents may be unavailable or unsuitable to provide consent for their son or daughter to participate in research. Based on consultation with local experts we clarify who can provide consent for the following adolescent groups:
- Adolescent under 18 living with his/her parents – Mother or father
- Married adolescent under 18 – Husband or wife (only if aged 18 or over), mother, father, mother-in-law, father-in-law.
- Adolescent under 18 not living with their parents: Adult appointed as the adolescent's caregiver (e.g. sibling aged 18+, aunt, uncle, grandmother, grandfather)
- Adolescent under 18 staying away from home for study or work – Mother, father, hostel warden
- Orphaned adolescent – Adult responsible for adolescent's care (e.g. aunt, uncle, grandmother, grandfather, sibling aged 18+), orphanage director

Developing appropriate, relevant recruitment documents for participants in Nepal
In my previous project in Nepal with TPO Nepal, approved by KCL REC (HR-18/19-8427), we discussed this issue with Annah Whyton, Research Ethics and Governance Manager until recently. Notably, KCL's information sheet and consent form templates are for the highest standard of GDPR compliance. Not all information sheets and consent forms necessarily have to meet this standard because the lawful basis for the research is a task in the public interest. Whilst there is a need to recognise GDPR, the focus on UK regulation is not helpful or necessarily relevant to participants in a Nepali setting. We decided that having more locally relevant documents was essential so that participants could fully understand the study and provide informed consent. We have therefore amended the KCL templates and will provide a more simplistic information sheet, as well as an additional data protection and storage information sheet available to participants on request, that outlines all the legal information on data protection and storage. We have removed the website link to KCL's use of personal data statement because many of our participants will not have access to the internet. This information has been included in the data protection and storage sheet instead. We will ensure that even if participants are not informed of all the GDPR aspects, the research team is acting in a way that is GDPR compliant regarding data storage. Information sheets and consent forms uploaded with this application are based on the approved documents we used successfully in HR-18/19-8427 and HR/DP-21/22-27152, and are therefore tried and tested with adolescents and the community in Nepal. In line with advice from Clare Heard (RGO), we have edited information sheets to clarify that this is a KCL sponsored project and that KCL and TPO are joint data controllers. TPO Nepal have approved the participant-facing documents.

Contact with adolescents and children
The study team will have regular contact with adolescents. The Nepal ethics committee do not require researchers working with children to have the equivalent of a Disclosure and Barring Service (DBS) check, but TPO Nepal have their own robust safeguarding policy, training, and reporting procedures in place. Dr Rose-Clarke underwent a DBS check in 2020 whilst at KCL.

Section C: Participants

C1 Detail your projected number of participants and provide justification for this sample size.
Please note: For projects involving mixed methods and/or multiple participant groups, you should provide an estimate of the number of participants taking part in each method.

Implementation of the intervention
Sports coaching practice (with smaller groups of adolescents): Practice activities will be conducted with groups of 10-20 adolescents (~100 total) in communities that are not study clusters. We will not collect data from these adolescents.

Sports coaching sessions: These will be open to all adolescents living in the study clusters. Based on our formative research we anticipate 40-60% of adolescents in each of the intervention clusters will attend the sports coaching sessions (128-192 adolescents in total).

Anyone from the community of 1000 people will be welcome to watch the community theatre performances and the sports mela. We will not be collecting data from community members at these events.

Endline survey
Participants will be all the adolescents living in intervention and control clusters = ~640 adolescents in total. Most of these participants will have participated in the baseline survey. Some participants will have participated in the sports coaching and/or adolescent clubs.

Process evaluation
We aim to conduct 24 focus groups with adolescents, coaches, community mobilisers and caregivers. There will be 6-8 participants per discussion. We will also conduct two interviews with teachers. The discussions and interviews are listed below:
2 Focus group discussions with boys who participated in the intervention (age 12-15)
2 Focus group discussions with boys who participated in the intervention (age 16-19)
2 Focus group discussion with boys that didn't participate (sampled based on consultation with sports coaches)
2 Focus group discussions with girls who participated in the intervention (age 12-15)
2 Focus group discussion with girls who participated in the intervention (age 16-19)
2 Focus group discussion with girls that didn't participate (sampled based on consultation with sports coaches)
2 Focus group discussions with male coaches
2 Focus group discussions with female coaches
2 Focus group discussions with female assistant coaches
2 Focus group discussions with male assistant coaches
2 Focus group discussions with female caregivers
2 Focus group discussions with male caregivers
2 Interview with teachers

Justifying the sample size
For process evaluation FGDs samples sizes are based on: (i) the number of participants needed to represent all the different viewpoints and stakeholders, diversity of caste/ethic groups, ages (adolescents), and genders; (ii) our own experience in previous projects of what is feasible and optimal; (iv) the need to keep the sample small enough to facilitate meaningful participation.

For the endline survey a sample of 4 clusters (~ 640 adolescents aged 12-19) will enable us to have enough statistical power to test randomisation and survey procedures across a range of settings and estimate the intra-cluster correlation coefficient.

C2a What are the Inclusion Criteria? Where appropriate explain how you will screen your participants. *(The selection criteria should be clearly defined for multiple participant groups)*

Implementation of the intervention
There are no inclusion or exclusion criteria for the intervention activities and no one will be excluded. However, we will target adolescents aged 12-19 living in the study clusters.

Endline survey
Survey participants will be any adolescent aged 12-19 living in the study clusters.

Process evaluation
Focus group discussions with:
- Boys who participated in the intervention age 12-15 (2 discussions)
- Boys who participated in the intervention age 16-19 (2 discussions)
- Boys that didn’t participate (sampled based on consultation with sports coaches) (2 discussions)
- Girls who participated in the intervention (12-15) (2 discussions)
- Girls who participated in the intervention (16-19) (2 discussions)
- Girls that didn’t participate (sampled based on consultation with sports coaches) (2 discussions)
- Male coaches delivering the intervention (2 discussions)
- Female coaches delivering the intervention (2 discussions)
- Female assistant coaches delivering the intervention (2 discussions)
- Male assistant coaches delivering the intervention (2 discussions)
- Female caregivers (one in each of the intervention clusters) (2 discussions)
- Male caregivers (one in each of the intervention clusters) (2 discussions)

Also, two interviews with secondary school teachers who have been involved in the intervention

C2b What are the Exclusion Criteria? Where appropriate explain how you will screen your participants. *(The selection criteria should be clearly defined for multiple participant groups)*

Implementation of the intervention
There are no inclusion or exclusion criteria for the intervention activities and no one will be excluded. However, we will target adolescents aged 12-19 living in the study clusters.

Endline survey
Adolescents who do not live in the study clusters (e.g. adolescents who live outside the clusters but are visiting friends/family in the study area at the time of the survey).

Process evaluation
N/A

C3 What are the upper and lower age limits? Provide justification for these where appropriate.

Adolescents will be 12-19 years which covers all ages in the Nepali secondary school system.
Assistant coaches will be youth aged 15-24 as defined by the World Health Organization, because they will have the maturity and skills to facilitate and support intervention activities, yet be close enough in age to relate to adolescents.
There are no upper or lower age limits for other participants in the study.

C4 How will potential participants be identified and approached?
Please note: If different recruitment methods will be used for participant groups, each group should be separately addressed.

We have detailed how each group of potential participants will be identified and approached in the "Recruitment process" doc uploaded in Section I.

C5 Do you have a current or prior relationship with any potential participants? (This includes professional and/or personal relationships)

- [ ] Yes, I do have a current or prior relationships with potential participants.
- [x] No, I do not have any current or prior relationships with potential participants.

C6 Gatekeeper Permission: Will you require an individual or organisation to grant you permission to approach/ access your intended participants? This includes gatekeepers contacting participants on your behalf

- [x] Yes, I will be using a gatekeeper to access potential participants
- [ ] No, I will not be using a gatekeeper to access potential participants

C6a Will the gatekeeper be in a position of influence or authority over the participants?

- [x] Yes, the gatekeeper is in a position of influence or authority over participants
- [ ] No, the gatekeeper will not be in a position of influence or authority over participants

C6b Outline who the gatekeeper is and how they will be used to facilitate recruitment.
Please note: Participants must only be approached once appropriate gatekeeper permission has been obtained.

We have permission from the municipality to conduct the research. We will obtain permission from the bolmunsa (village headman) to conduct the research in each of the clusters and from the school principal as needed.

C6c Please outline how gatekeeper permission will be obtained

Members of the local study team will organise a time to visit the bolmunsa and school principal to explain the study and obtain their permission.

C6d Explain how you will mitigate any pressure to participate that may be felt by potential participants as a result of the gatekeepers position.

We will explain to potential participants at meetings and reiterate in the information sheet that the research/intervention is not part of the school curriculum, there is no pressure to participate, and that they will not be disadvantaged if they decide not to participate.

C7 Please specify any incentives being offered and a justification for their use.

We will cover transportation costs incurred by adolescents and adults participating in the interviews and focus group discussions.
They will also be offered refreshments at the start of the activity (juice and biscuits).
No incentives will be offered to adolescents or their families participating in the endline survey or intervention activities.

Section C: Informed Consent

C8 Will informed consent be sought from all participants? [x] Yes [ ] No

C8a How will informed consent be obtained for each data collection method/participant group? Who will take consent and how will it be recorded?

Note: Justification must be provided if you will not be providing all participants with an information sheet and gaining written consent. ***Please see the guidance icon for further information on tailoring informed consent processes.***

The Nepal Health Research Council (NHRC) Ethics Committee requires caregiver consent and adolescent assent for all adolescents under the age of 18, which we will adhere to in this study. TPO Nepal's 17 year's of experience conducting mental health research in Nepal has found that it is better to give just one information sheet and consent form (rather separate versions for parents/caregivers and adolescents) because adolescents are often more educated and more literate than their parents, and both groups find it less confusing to have one form. Where possible participants will be given at least 24 hours to decide if they would like to participate but this may not always be possible (see Section C9).

Implementation of the intervention: We will only collect anonymous data in intervention activities, e.g. total number of adolescents attending by caste/ethnic group, age group and gender; topics/activities covered by the facilitator in the session. However, we will still collect informed consent from the adolescent and caregiver, where appropriate, as described in the "Recruitment Process" doc for their participation. The intervention will be explained to potential participants and their parents by members of the intervention team at meetings held in the community and school (see Recruitment Process doc). It will be made clear that there is no pressure to participate because the intervention is not part of the school curriculum and they will not be disadvantaged in any way.

Endline survey
A trained researcher will explain the study to the parent/caregiver and adolescent and give them a consent form and information sheet (in Nepali). The researcher will ask if they have any questions and make sure participants/caregivers are satisfied before providing written consent. We will stress that just because the adolescent participated in the baseline survey and/or the intervention activities they are not obliged to participate in the endline survey.

Process evaluation
Adolescents interested in participating will be given a consent form and information sheet (in Nepali) by a researcher, which they can take home and read with their parent/caregivers. Adolescents and parent/caregivers will be able to ask researchers any questions they have about the study either in person or by phone (contact details on the information sheet). Adolescents who return signed consent forms, signed by their parent/caregivers and themselves will be able to participate.

Adults (i.e. parents/caregivers, sports coaches, teachers and peer mentors) participating in FGDs and SSIs will be given an information sheet, and the researcher will verbally describe the study and what would be expected of them if they decided to participate. Adults will also be able to ask the researcher any questions.

C9 How long will participants be given to decide if they wish to participate?

Please provide justification if participants will be given less than 24 hours

As per our formative research and the baseline survey, for the most part we anticipate it will be possible to give participants more than 24 hours to decide. Participants for whom it may not be feasible include: adolescents living in remote villages that are difficult for the team to access (for example, some villages may not be accessible by road and researchers may have to trek to reach them); participants who, because of travel or their schedules have limited availability. It is important to represent these participant groups in the study. Therefore, if it is not possible to give participants at least 24 hours we will discuss with them arranging the interview/FGD for later on the same day; making sure they have plenty of time to ask questions about the study; giving them chance to discuss the study with others (e.g. a husband, an older sibling, colleague, etc); and emphasising that they are under no pressure to participate.

C10 Detail the process by which participants may withdraw from the research both during the research and after it has been completed. A final withdrawal date should also be provided, after which participants may no longer withdraw their data from the study.

We will explain and emphasise to participants that they have the right to withdraw from the study without providing a reason. In the information sheet, we will inform participants that they have the right to withdraw their data up until one month after their participation, however it is not possible to remove an individual contribution from a focus group discussion due to the difficulty in isolating individual contributions and the fact that doing so if this were possible would remove valuable context to others' contributions.

Section D: High Risk Research

D1e Risk Identified: Participation in this research may identify urgent mental health risks, including, but not limited to, suicidal ideation and/or self-harm intent:

i) Explain how urgent mental health concerns could be identified as part of this study.

Participants may disclose suicidal ideation upon answering questions about mental health in the endline survey. Sports coaches, teachers, and assistant coaches may come to know of high risk adolescents participating in the intervention activities.

D1e(iii) What steps will be taken to mitigate any potential risks in response to the identification of urgent mental health concerns?
Please review the guidance icon before completing your response.

Endline survey participants
Participant information sheets will outline the topic (mental health promotion) and, where applicable, mention that participants will be asked about mental health problems. Researchers will be trained to ask questions in a sensitive and culturally respectful way, and to conduct interviews/focus group discussions in a private space where participants feel comfortable. Participants will be reminded that they can stop or pause if they are uncomfortable, and that they don't have to answer questions if for any reason they do not want to. If participants do become visibly distressed, researchers will ask if they would like to take a break or finish and will follow up with them individually. If participants disclose suicidal ideation, the researcher will activate a standard operating procedure (used in our previous projects with TPO Nepal, also approved by KCL REC) which involves alerting the participants' parents/caregivers and arranging an urgent appointment with the TPO Nepal psychosocial counsellor who is based in the study area (please see "SMART Adverse Events Reporting Procedure" doc in Section I. They will also be signposted to relevant support services as appropriate. Cases will be documented and submitted to the project coordinator for review.

Adolescents participating in the intervention activities
Any disclosure of suicidal ideation to a coach, teacher or assistant coach will trigger a standard operating procedure (as above). Staff will be trained to use this procedure to first assess the patient's risk and refer as necessary to the TPO Nepal psychosocial counsellor who is based in the study area, informing senior members of the study team. Cases will be documented as above and referred to relevant support services if appropriate.

D2 If there are any additional risks or burdens to participants that have not been addressed above, please provide further details and explain how these risks will be mitigated:

Our Nepali/English research team has extensive experience interviewing and implementing mental health research in rural Nepali contexts and we have been working in Bardiya on the formative work for this project since November 2021. TPO Nepal has been working with the Nepal Health Research Council to conduct research of the highest ethical standards since 2005. Research assistants receive training in quantitative and qualitative data collection including consent procedures, how to maintain confidentiality and data protection.

COVID-19 - The pandemic continues to affect Nepal, though there are currently no restrictions in place. We will be alert and responsive to changes in Nepali and UK rules and guidance, specifically information and guidance from the Nepal Health Research Council. We will encourage members of the research team to observe social distancing and hand washing recommendations, wear a mask, and conduct research activities outdoors where appropriate.

If any adolescents incur an injury due to the intervention (e.g. on the football pitch) coaches, teachers and/or community mobilisers will activate a standard operating procedure that involves: assessment of severity, consultation with the adolescent and their family, support to access appropriate health facilities where necessary, e.g. the primary health care centre or hospital. Coaches will have access to a first aid kit and receive basic first aid training.

D3 What are the potential benefits to the participant?

Communities in the intervention arm will benefit from access to sports coaching. We will also provide sports equipment to the local municipality.

D4 Will participants be guaranteed complete anonymity in the final report and any further research output/s?

- (selected) Yes
- No

D4a Please explain how you will ensure participants remain completely anonymous in the final report or any other research output/s.

> We will not use any other identifying information about participants in our research outputs. Personal names, names of schools, or location information will all be removed from quotes used to report qualitative findings. Quantitative data will be pseudonymised prior to analysis and reported findings will not identify any individual participants.

Section E

E1 Does the project involve the collection and/or use of personally identifiable information (as outlined in UK GDPR)?

Identifiable information is data that can be used to identify an individual, either directly (such as full name, address, Twitter handle, etc) or indirectly through the combination of several pieces of data. The most common examples are names, contact details, audio/ video recordings, usernames etc. However, data that has the potential to indirectly identify a participant should also be treated as identifiable.

Please see the guidance icon for more examples of when data should be considered identifiable or contact the Research Governance Office: rgo@kcl.ac.uk

Please indicate which of the following applies:

- (selected) Yes, the project involves the collection and/or use of identifiable information
- No, I will not be collecting and/or accessing any identifiable information for this project.

***Before completing the following questions, please ensure you have read the KCL Research Data Management Guidelines* and guidance on the UK General Data Protection Regulation (UK GDPR)**

Section E (I): UK Data Protection Requirements

E2 Who is the Data Controller? Please see the guidance icon for a definition of a Data Controller

- King's College London
- An External Individual/ Institution
- (selected) King's College London is a Joint Data Controller with an External Individual/ Institution

E2a Details of the external Data Controller

Name	Dr Nagendra Prasad Luitel
Institution	TPO Nepal
Email	luitelnp@gmail.com / npluitel@tponepal.org.np

E3 Please state which of the following categories of personal data (relating to research participants) will be collected, processed or stored ***at any stage*** of the research project? (please select all that apply):

- [x] Name and/or signature (this includes those recorded on consent forms)
- [x] Date of Birth/ Age
- [x] Contact Details (email address, phone number, etc)
- [x] Identification Number (participant number, NHS number, staff number)
- [x] Location Data (full address, postcode, IP address etc)
- [ ] Online Identifier (identifiers provided by devices or apps, cookies etc)
- [x] Identifiable Image or Recording (photographs, video recordings and audio recordings) including interview recordings
- [x] Biographical Data (includes gender, marital status, employment history/job, etc.)
- [ ] Other

E4 Will any of the following special categories of personal data (relating to research participants) be collected, processed or stored at any stage of the research project from this point forward? (please select all that apply):

- [x] Race and/or Ethnic origin
- [ ] Political opinions
- [x] Religious or philosophical beliefs
- [ ] Trade Union Membership
- [ ] Processing of genetic data
- [ ] Biometric data for the purpose of uniquely identifying a natural person
- [x] Health data
- [ ] Sex life
- [ ] Sexual orientation
- [ ] Criminal convictions or offences
- [ ] None of the above

E5 The UK GDPR identifies research as a 'public task' and as such you are advised to use 'public task' as your lawful basis for processing personal data. As you will also be collecting special category data, you are also required to state a condition for processing this data. As a KCL researcher, you are advised to use the 'archiving, research and statistics' condition for processing special category data.

Please confirm you will be processing personal and special category data under the 'Public task' and 'Archiving, research and statistics' lawful bases

(•) Yes, I will be processing personal data under the 'Public task' and special category data under the 'archiving, research and statistics' condition for processing

( ) No, I will be processing data under an alternative lawful basis and/or condition for processing

Section E (II) Data Handling, Protection and Storage during data collection & analysis

E6 In which format/s will the personal data be stored in while data collection and analysis is ***ongoing***?

- [x] Electronic Format
- [x] Hard Copy

E6a Electronic format - select all that apply:

Please note, where possible a KCL storage option should be selected in addition to any external storage

- ☐ KCL network drive
- ☐ KCL SharePoint
- ☐ NHS Network Drive
- ☐ KCL OneDrive
- ☐ Rosalind
- ☐ External hard drive
- ☐ USB
- ☐ KCL laptop
- ☐ Personal laptop
- ☑ Other

If other, please specify:

All data collection is being conducted in Nepal by TPO Nepal employees who have no employment affiliation with KCL. KCL's role is that there is a KCL co-PI (Dr Kelly Rose-Clarke) who will be receiving de-linked pseudoanonymised datasets which will be analysed at KCL and stored on KCL servers. Dr Joanna Morrison, a co-I at UCL) will also be receiving de-linked pseudoanonymised data which she will analyse.
TPO Nepal researchers will collect quantitative data from participants using mobile phones and/or tablets, owned by TPO Nepal, which will be password protected and encrypted where possible. They will unlink the identifiers from the dataset using unique participant IDs. The dataset containing identifiers will be stored on a password-protected file on TPO's secure central server.
TPO Nepal researchers will make audio recordings on voice recorders. Recordings will be uploaded to the TPO server (and deleted from the recorder) and deleted from the server once they have been transcribed or, where transcription is not required (e.g. transect walks, cognitive interviews), after detailed notes have been taken. Transcriptions and notes will be stored on the TPO Nepal server.
Any paper data (e.g. hand-written notes) will be stored in a locked filing cabinet at the TPO Nepal office.

E6a i) Can you confirm that the storage device is encrypted and, where possible, password protected?

- ○ Yes
- ◉ No

If no, please explain how data security is ensured:

All personal data will be stored at TPO Nepal and not shared with KCL. Dr Rose-Clarke (PI) will only receive de-linked pseudoanonymised datasets, transferred using a secure file transfer and storage service.
Across their various research projects, TPO Nepal ensure data security by storing datasets on their internal server, and using password protected devices (phone/tablet) to collect data. Their standards of data security meet and exceed data governance and ethical requirements in Nepal.

E6b Hard copy - select all that apply:

- ☐ Stored securely within the College
- ☑ Secure repository when in the field
- ☐ Stored securely on NHS premises
- ☐ Other

Please provide details of the specific location:

Paper copies of consent forms, SSI/FGD notes, etc will be kept in a locked cabinet in the TPO office.

E7 Data Access: **During data collection & analysis**, will data be shared with any researcher or individual <u>outside of the immediate research team</u>? (*Please note this includes sharing any audio/ video recordings with transcription services)*

- ○ No, data will not be shared with any other researcher or individual outside of the immediate research team during data collection and analysis
- ◉ Yes, data will be shared with another researcher or individual outside of the immediate research team during data collection and analysis (for example, for the purposes of transcribing the data)

E7a Select which of the following third parties data will be shared with **during data collection and analysis**:

- ☐ Third party transcription service
- ☐ Other third party private organisation (includes commercial companies)
- ☑ Third party public organisation (includes universities)
- ☐ Third party charity organisation
- ☐ NHS
- ☐ Funder
- ☐ Other

E7a i) Please outline who will have access and why this is necessary

Co-I, Dr Joanna Morrison at UCL, will be working on qualitative data analyses and will receive pseudoanomysed de-linked data. A consultant statistician (KCL) and health economist (UCL) will also be analysing the pseudonymised de-linked data. There will not be any third parties receiving personal data.

E7b Will data be shared outside of the UK?

- ◉ Yes
- ○ No

E7b i) Please state who data will the shared with and the country in which they are located

Data will be collected in Nepal and pseudoanonymised unlinked datasets will be shared with members of the research team in the UK.

E7c Will data be shared in an identifiable format? (*Please note this includes any audio/ video recordings)*

- ○ Yes
- ◉ No

Please note: Data (either in an identifiable or anonymous format) should not be shared with external third parties unless there is an appropriate agreement in place. For guidance please contact the Contracts Team.

E8 Once **data analysis is complete** how will research data (including any participant contact details) be stored:

- ○ Data will be stored in an identifiable format after analysis
- ◉ Data will be pseudonymised after analysis
- ○ Data will be fully anonymised immediately after analysis

E8a Please outline how you will pseudonymise each category of personal data as selected under E3 & E4.

> Data will be unlinked and identifying information will be securely retained by TPO Nepal.

Section E (III) Data Handling, Protection and Storage on completion of the research

E9 In which format/s will the personal data be stored following ***completion*** of data collection and analysis?

- [x] Electronic Format
- [ ] Hard Copy

E9a Electronic format - select all that apply:

Please note, where possible a KCL storage option should be selected in addition to any external storage

- [ ] KCL network drive
- [x] KCL SharePoint
- [ ] NHS Network Drive
- [ ] KCL OneDrive
- [ ] Rosalind
- [ ] External hard drive
- [ ] USB
- [ ] KCL laptop
- [ ] Personal laptop
- [x] Other

If other, please specify:

> Pseudoanonymised unlinked data will be stored on TPO Nepal's password protected secure server.

E9a i) Can you confirm that the storage device is encrypted and, where possible, password protected?

- ○ Yes
- ◉ No

E9a ii) If no, please explain how data security is ensured:

The server is controlled by TPO Nepal who ensure data security through password-protected accounts for individual employees.

E10 Expected date that the data (including any participant contact details) will no longer be stored in an identifiable/pseudonymised format:

Please note: Data should only be stored in an identifiable format for as long as is absolutely necessary.

01/01/2025

E10a Please provide a justification to why personal data will be stored for the length of time indicated:

Beyond the study it is possible that some participants will continue to be supported by TPO Nepal (for example if they are referred to the psychosocial counsellor) in which case personal information will be retained to facilitate this.

E11 Data Retention Schedule

Research data should be stored in line with the KCL Data Retention Schedule. Please note that raw data should be stored in an ***anonymous/ pseudonymous*** format where possible.

☑ I confirm that research data will be stored in line with the KCL Data Retention Schedule

E12 Data Access: Please confirm that no other researcher or individual outside of the immediate research team will have access to any personal data on completion of data collection and analysis

◉ Yes, I confirm that no other researcher or individual outside of the immediate research team will have access to any personal data on completion of data collection and analysis

○ No, another researcher or individual outside of the immediate research team will have access to personal data on completion of data collection and analysis

Section E (IV): Publication & Data Sharing on completion of the research

E13 Will any data from which participants could be identified be published (this could be direct quotes or biographical data that could lead to the identification of an individual)?

○ Yes

◉ No

E14 Will research data be shared with any external third parties **after data analysis is complete**?

◉ Yes

○ No

E14a Select which of the following third parties data will be shared with:

- ☐ External Project Supervisor
- ☐ Third party private organisation (includes commercial companies)
- ☐ Third party public organisation (includes universities)
- ☐ Third party charity organisation
- ☐ NHS
- ☐ Funder
- ☑ Other

If other, please specify:

Other researchers who wish to analyse the data may request access. Requests will be reviewed on an individual basis by the Principal Investigators. Data information sheets will inform participants that their anonymised data may be shared for this purpose.

Please note: Data (either in an identifiable or anonymous format) should not be shared with external third parties unless there is an appropriate agreement in place. For guidance please contact the Contracts Team.

E14b Will data be shared in an identifiable format? (*Please note this includes any audio/ video recordings)*

- ○ Yes
- ◉ No

E15 Will data be archived for further use?

- ◉ Yes
- ○ No

E15a Will the archived data contain identifiable information?

- ○ Yes
- ◉ No

E16 Research Dissemination: How will results be disseminated?

- ☐ Internal report (thesis)
- ☑ Journals
- ☑ Conference
- ☑ Other

If other please specify:

Dissemination workshops, policy briefs, website summaries, community meetings.

Section H: Insurance, Risks and Ethical Issues

H1 Does the project involve any of the Risk Assessment criteria outlined in the information icon guidance? (x) Yes ( ) No

H1a I confirm that I will complete a Risk Assessment Form which will be signed by my Supervisor or Head of Department prior to commencing data collection (x) Yes ( ) No

Please note: Your department should be able to provide you with a Risk Assessment Form. If they are unable to do so, please contact Health and Safety Services for further advice.

H2 Project Insurance Cover - Please indicate if your project involves any of the following -

- [ ] An overseas clinical trial
- [ ] Recruitment of overseas healthcare patients
- [x] A physical or mental health intervention involving human subjects (see guidance icon for definition)
- [ ] None of the above

H2a I confirm that I have read the exclusion criteria for the College's Clinical Trials and Research Projects Involving Human Subjects Insurance Policy, detailed in the guidance icon, and that:

( ) a) This project does not fall under the exclusion criteria of the policy

(x) b) This project falls under the exclusion criteria and I have gained approval from the Finance Department, as instructed in the guidance icon

( ) c) This project falls under the exclusion criteria but approval has not been granted by the Finance Department

H2b Please state which exclusion criteria applies

Any overseas trial.

H3 Travel Insurance for overseas studies: I confirm that my travel insurance arrangements are as follows:

(x) a) I will secure College travel insurance (see guidance icon for further details)

( ) b) I will secure personal travel insurance

( ) c) I do not require travel insurance as I will conduct the research in my country of legal residence

( ) d) I will not secure travel insurance for overseas travel

H4 I confirm that if Disclosure & Barring Service clearance is required for my study, this will be obtained prior to the commencement of data collection. (x) Yes ( ) No ( ) N/A

H5 I confirm that the No Fault Compensation Scheme will be offered to all UK based participants. (x) Yes ( ) No

H6 Give the details of any other review body approvals or permissions obtained (including other Ethics Committees, peer review, R&D permission etc).

The Nepal Health Research Council have already approved the study (letter attached). KCL REC approved the first phase of the study (HR/DP-21/22-27152).

H7 Give details of any other ethical issues which have not been addressed elsewhere in the application and explain how you will mitigate these risks.

Section I: Supporting Documents

I1 Participant Information Sheet

Information Sheet templates can be found under 'Recruitment documents'.

Consent form (if applicable)

I2 Consent form (if applicable)

Documents

Type	Document Name	File Name	Version Date	Version	Size
Consent Form	SMART Information sheets and consent forms - implementation and endline	SMART Information sheets and consent forms - implementation and endline.docx	11/11/2022	2.0	197.7 KB

Questionnaire/Survey template/s

I4 Questionnaire/Survey template/s

Documents

Type	Document Name	File Name	Version Date	Version	Size
Questionnaires	Example_ado_endline survey	Example_ado_endline survey.xlsx	04/08/2022	1.0	39.6 KB

List of Indicative questions or topic guides

I5 List of Indicative questions or topic guides

Documents

Type	Document Name	File Name	Version Date	Version	Size
Interview guide	Process evaluation topic guides	Process evaluation topic guides.docx	04/08/2022	1.0	20.2 KB

Evidence of any other approvals or permissions (includes gatekeeper, R&D, other ethical approvals) (if applicable)

I6 Evidence of any other approvals or permissions (includes gatekeeper, R&D, other ethical approvals)

Documents

Type	Document Name	File Name	Version Date	Version	Size
Permission/Approval Letter	NHRC approval	NHRC approval.pdf	22/04/2022	1.0	724.3 KB
Permission/Approval Letter	KCL Approval letter	Approval letter.pdf	04/08/2022	1.0	108.0 KB

Approach letters to gatekeeper organisations (if applicable)

I7 Approach letters to gatekeeper organisations

Advertisement document (email, poster, flyer etc) (if applicable)

I8 Advertisement document (email, poster, flyer etc)

Cover Letter (for amendments and modifications) (if applicable)

I9 Cover Letter (for amendments and modifications)

Documents

Type	Document Name	File Name	Version Date	Version	Size
Other	Cover letter 01112022	Cover letter 01112022.docx	11/11/2022	1.0	77.9 KB

Other (if applicable)

I10 Other

Documents

Type	Document Name	File Name	Version Date	Version	Size
Other	SMART Adverse Events Reporting Procedure	SMART Adverse Events Reporting Procedure.docx	22/09/2022	1.0	122.4 KB
Other	Tania Pattenden email Aug 22	Tania Pattenden email Aug 22.pdf	11/11/2022	1.0	219.6 KB
Other	AERSQ-E Questionnaire (English) - new	AERSQ-E Questionnaire (English) - new.docx	11/11/2022	1.0	21.0 KB
Other	Recruitment process	Recruitment process.docx	11/11/2022	2.0	16.9 KB

Researcher/Applicant

J1 Researcher/Applicant Signature

I undertake to abide by accepted ethical principles and appropriate code(s) of practice in carrying out this study. The information supplied above is to the best of my knowledge accurate. I have read the Application Guidelines and clearly understand my obligations and the rights of participants, particularly as regards obtaining valid consent. I understand that I must not commence research with human participants until I have received full approval from the ethics committee.

Please note that in order to authorise your application you must sign off using your KCL email address i.e. joe.bloggs@kcl.ac.uk and your KCL password.

Signed: This form was signed by Kelly Rose-Clarke (kelly.rose-clarke@kcl.ac.uk) on 11/11/2022 1:29 PM
